# The Genetic Specificity of Cognitive Tests After Controlling for General Cognitive Ability

**DOI:** 10.1007/s10519-025-10213-5

**Published:** 2025-02-18

**Authors:** Francesca Procopio, Engin Keser, Jacob Knyspel, Margherita Malanchini, Kaili Rimfeld, Robert Plomin

**Affiliations:** 1 Social, Genetic and Developmental Psychiatry Centre, Institute of Psychiatry, Psychology and Neuroscience, King’s College London, London, UK; 2 School of Biological and Chemical Sciences, Queen Mary University of London, London, UK; 3 Department of Psychology, Royal Holloway, University of London, Egham, UK

**Keywords:** Cognitive tests, General cognitive ability, Genetic specificity, Genomic SEM

## Abstract

Diverse tests of cognitive abilities correlate about 0.30 phenotypically and about 0.60 genetically. Their phenotypic overlap defines general cognitive ability (g), driven largely by genetic overlap. Consequently, much of our understanding of the genetic landscape of specific cognitive tests likely reflects g rather than the tests themselves. Removing this g-associated genetic variance will sharpen research on cognitive tests. Here, we use Genomic Structural Equation Modelling (Genomic SEM) to remove shared genetic variance among 12 diverse cognitive tests that capture verbal and nonverbal cognitive domains. We applied Genomic SEM to summary statistics from the largest genome-wide association studies of verbal tests (GenLang Consortium, five tests) and largely nonverbal tests (UK Biobank, seven tests) to chart the genetic landscape of the 12 tests independent of g as compared to uncorrected cognitive tests. We found that SNP heritabilities were nearly as high for the tests corrected for g as uncorrected: the average SNP heritability was 0.16 (SE = 0.02) for the uncorrected tests and 0.13 (SE = 0.02) for the tests corrected for g. Despite this, the genetic landscape of the cognitive tests transformed after controlling for genomic g. The matrix of positive genetic correlations for the cognitive tests (average 0.45) disappeared after g-correction, and some strong negative correlations emerged; for instance, Memory and Word (-0.72), Fluid and Symbol (-0.72), and Tower and Spelling (-0.79). The summary statistics for these g-corrected cognitive tests can be used by researchers to create polygenic scores that focus on the specificity of the tests.

**Supplementary Information:**

The online version contains supplementary material available at 10.1007/s10519-025-10213-5.

## Introduction

In 1904, Charles Spearman observed a phenomenon that has since become one of the most consistently replicated and widely accepted findings in psychology: positive correlations, known as a positive manifold, among tests of specific cognitive abilities. This led him to hypothesize that a common factor, general cognitive ability (g), accounts for the shared variance (Spearman 1904). Later, Spearman introduced the concept of specific factors, which represents the unique unshared component of group factors, such as spatial and reasoning abilities (Spearman 1927). Here we focus not on group factors of specific cognitive abilities (SCAs) but on individual cognitive tests independent of g.

Not only do cognitive tests show substantial phenotypic correlations, about 0.30 on average (Carroll 1993), but they also demonstrate even higher genetic and genomic correlations, typically greater than 0.60 (Plomin and Deary 2015; Grotzinger et al. 2019; Hindley et al. 2023; Rimfeld et al. 2015; Trzaskowski et al. 2013). These correlations indicate that the positive manifold among cognitive tests is largely driven by genes. Although the magnitude of genetic correlations among diverse cognitive tests is remarkable, the fact that genetic correlations are considerably less than 1.0 provides evidence for genetic specificity.

Due to the pervasive influence of g, much of our understanding of the genetic architecture of cognitive tests reflects the genetic variance that is shared by all tests, rather than variance specific to each test. In fact, genetic studies often leverage this positive manifold and combine multiple cognitive traits to increase statistical power, and to focus on shared genetic variance rather than unique genetic variance. The hypothesis underlying the present research is that removing the effects of g will sharpen genetic research on the specificity of cognitive tests.

Despite the widespread influence of g, there have been few genetic or genomic studies of cognitive tests independent of g. The few available twin studies indicate that g-corrected cognitive tests remain substantially heritable despite controlling for the heritability of g (Procopio et al. 2022; Rimfeld et al. 2015, 2017; Tosto et al. 2013). Multivariate genetic twin analyses imply genetic specificity because genetic correlations among cognitive tests are less than 1.0, but none have directly investigated the genetic relationships among cognitive tests independent of g. However, a novel application of network analysis to multivariate twin data has recently yielded the intriguing finding that some of the typically positive genetic correlations between pairs of cognitive tests switch to become strongly negative genetic correlations when the network of relationships with all other tests is partialled out (Knyspel and Plomin 2024). This finding implies that some DNA variants might operate in opposite directions for pairs of g-corrected cognitive tests, which would obfuscate research on specific cognitive abilities such as genome-wide association analyses.

Genome-wide genotypes offer new opportunities to investigate changes in the genetic landscape before and after controlling for g (Procopio et al. 2024). The only relevant genome-wide association (GWA) analysis conducted analyses that are comparable to GWA of g-corrected cognitive tests investigated English, mathematics and science achievement phenotypically corrected for g (Donati et al. 2021). Although these subjects are educational outcomes, they are conceptualized as measures of cognitive abilities in this context due to the high phenotypic and genetic correlations between cognitive abilities and educational performance. Despite being underpowered (*N* = 3,000), the study found significant SNP heritabilities for residualized mathematics and science, and one GWA-significant SNP was found across the analyses for science. Genomic investigations of g-corrected cognitive tests with larger sample sizes are needed to further investigate the genetic architecture of specific cognitive traits independent of the influence of generalist genes. In lieu of sufficiently large GWA analyses of g-corrected cognitive tests, the aim of our study was to leverage Genomic Structural Equation Modelling (Genomic SEM) (Grotzinger et al. 2019) to investigate the genomics of cognitive tests independent of g. We modeled GWA summary statistics from the largest GWA association studies of five verbal tests (Eising et al. 2022) and seven tests that are primarily nonverbal (de la Fuente et al. 2021). Combining twelve summary statistics from these two GWA studies will produce a better representation of g, which includes both verbal and nonverbal abilities.

Unlike previous studies, we used Genomic SEM to remove the genetic influence of g from each of the 12 tests and created 12 sets of GWA summary statistics. Our goal was to compare the genetic correlational landscape of cognitive tests before and after controlling for g, as well as to compare downstream analyses of GWA-significant SNPs, SNP heritability, and genetic correlations with other cognitive traits and traits outside the cognitive domain. This study was preregistered on the Open Science Framework (OSF; https://osf.io/9qwbn/); deviations from the registered protocol are described in the Supplementary Note.

## Methods

### Samples and Measures

In order to comprehensively investigate the genetic landscape of cognitive tests, we selected GWA summary statistics for 12 tests from two studies: de la Fuente et al. (2021), which included seven largely nonverbal performance tests, and Eising et al. (2022), which analyzed five reading and language tests. This combination allowed us to construct a broader g factor that incorporates both verbal and nonverbal components. Although reading and language tests are often referred to as educational skills, we define specific cognitive abilities more broadly to include not only traditional psychometric tests such as reasoning, memory and perceptual speed but also tests traditionally considered as educational outcomes such as spelling, phoneme awareness and reading words and nonword.

The first study (de la Fuente et al. 2021) conducted a GWA analysis of cognitive tests from the UK Biobank (UKB; Sudlow et al. 2015). The UKB is one of the world’s largest biobanks; from 2006 to 2010, it recruited half a million individuals aged 40 to 69 across the UK. de la Fuente et al. used genotypic data from UKB participants of European ancestry (*N* = 11,263 to 331,679 adults aged 40 to 75). Participants were included if they completed at least one of seven cognitive tests: Matrix Pattern Recognition (a measure of nonverbal reasoning, which we refer to as ‘Matrix’), Pairs Matching Test (episodic memory, ‘Memory’), Reaction Time (perceptual motor speed, ‘Reaction’), Symbol Digit Substitution (information processing speed, ‘Symbol’), Trail Making Test-B (executive function, ‘Trail’), Tower Rearranging (executive function, ‘Tower’), and Verbal-Numerical Reasoning (fluid intelligence, ‘Fluid’). Due to length restrictions in testing such a large sample, the UKB tests are at the lower end of reliability for cognitive tests (Fawns-Ritchie and Deary 2020).

The second study (Eising et al. 2022) conducted a meta-analytic GWA analysis using data from the international Genetics of Language consortium (GenLang), focusing on speech, language, reading and related verbal skills. We used summary statistics from a meta-GWA analysis of 22 independent cohorts of individuals of European ancestry (*N* = 13,633 to 33,959 children and young adults aged 5 to 26 years). GWA summary statistics were available for five tests:Word Reading (number of words read aloud correctly, ‘Word’), Nonword Reading (nonwords are phonemes that look like words but have no meaning, ‘Nonword’), Spelling, Phoneme Awareness (‘Phoneme’), and Nonword Repetition (number of nonwords repeated aloud correctly, ‘Repetition’).

Scores on all tests were coded so that higher scores represented better (faster or more accurate) performance. Further information on the measures, cohorts and data collection procedures of both studies is included in the Supplementary Note and the original publications.

### GWA Analysis of g-Corrected Cognitive Tests Using Genomic Structural Equation Modelling

To create summary statistics for the 12 cognitive tests residualized by their genomic covariance, genomic g, we used Genomic Structural Equation Modelling (SEM) in R studio (Grotzinger et al. 2019). Genomic SEM combines SEM with factor analysis to explore the genetic relationship among complex traits. After constructing a genetic covariance matrix (S) along with its corresponding sampling matrix (V), the SEM of choice is fit to the genetic covariance matrix, and the parameters and their standard errors (SE) are estimated.

**Fig. 1:**
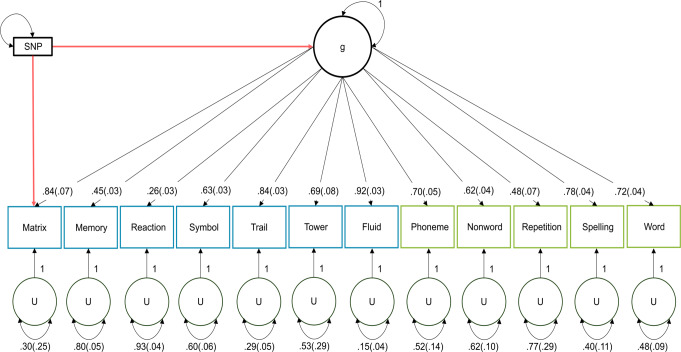
Common factor model. *Note.* Rectangles represent the observed variables, and the circles represent the latent variables extracted from the measured data. The one-headed arrows represent standardized factor loadings, which can be interpreted as standardized regressions between the predictor and the outcome the arrow is pointing to. The two-headed arrows represent the residual variances of the variable. Standard errors (SE) are shown in parentheses. The first 7 observed variables in blue represent the tests from UKB, and the second 5 observed variables in green represent the tests from GenLang. The two red arrows from SNP to g and Matrix represent the Genomic-SEM model we fit to construct GWA summary statistics of Matrix independent of genomic g. We ran the model 12 times, once for each test, resulting in 12 g-corrected cognitive test summary statistics

We created g-corrected cognitive test GWA summary statistics by fitting the model shown in Fig. 1 to the S and V matrices. This model extracted a single common factor, genomic g, from the 12 GWA summary statistics, while simultaneously residualizing the effect of genomic g from each test. We ran the model 12 times, correcting each test for genomic g individually. For more details on the procedure, see the Supplementary Note.

We extracted a single common factor, the ‘g factor’ (Jensen 1998), as a conservative approach that captures as much covariance as possible among the 12 cognitive tests, rather than removing covariance that clusters more tightly, for example on verbal and nonverbal factors. In other words, our goal is not to identify the best-fitting model of cognitive abilities, which involves a hierarchical model with verbal and nonverbal group factors, but rather to assess the specificity of the cognitive tests independent of a first principal component that represents what the tests share in common. It should also be noted that our index of g is dependent on the 12 tests that are available from GWA analyses. No battery of tests can provide a perfect index of g, although the seven UKB tests and the five GenLang tests represent a broader set of nonverbal and verbal tests than previously available. Moreover, as long as a diverse battery of tests are analyzed, Spearman (1927) hypothesized ‘indifference of the indicator’ when it comes to assessing g, an hypothesis validated in empirical analyses showing correlations of 0.99 between g factors derived from different test batteries (Johnson et al. 2004).

### SNP Heritabilities and Genetic Correlations

We used LD Score Regression (LDSC) in Genomic SEM to estimate SNP heritabilities and genetic correlations from the GWA summary statistics (Bulik-Sullivan et al. 2015; Grotzinger et al. 2019) for the 12 cognitive tests uncorrected and corrected for g. We focused on the comparison between genetic correlations among the g-corrected and uncorrected cognitive tests. Given the broad and diverse association of cognitive traits with important life outcomes, we also used LDSC in Genomic SEM to investigate genetic correlations between the GWA summary statistics for the g-corrected and uncorrected cognitive tests and 64 external GWA summary statistics across seven domains: Behavioral; BMI, height and weight; Cognitive; Mental Health; Personality; Physical Health; and Socio-economic status (SES) traits (see Supplementary Table S1 for a full list).

### GWA Significant Hits and Functional Mechanisms

We used the web-based platform Functional Mapping and Annotation of Genome-wide Association Studies (FUMA) to further explore GWA summary statistics for g-corrected cognitive tests (Watanabe et al. 2017). This included identifying GWA-significant SNPs and analyses of gene sets and gene properties using Multi-marker Analysis of GenoMic Annotation (MAGMA) within FUMA. Gene set analysis was used to identify biological pathways and processes which are associated with the g-corrected cognitive test GWA summary statistics, whereas gene-property analysis explored potential tissue-specific effects of SNPs by analyzing specific tissue types (e.g. the hippocampus). For further information on the methods and results of these analyses, see the Supplementary Note and Supplementary Tables S6-S26.

## Results

### GWA Analysis of 12 Cognitive Tests Uncorrected and Corrected for g Using Genomic SEM: Evidence for both Commonality and Specificity

To investigate the unique genomics of the 12 cognitive tests, we used Genomic SEM to fit a single common factor model to the 12 cognitive tests. The full model parameter estimates are in Fig. 1 and fit indices are in Supplementary Table S2. All 12 tests load significantly on genomic g – the loadings range from 0.26 for Reaction Time (Reaction) to 0.92 for Verbal-Numerical Reasoning (Fluid) (UKB) (mean = 0.66; SE = 0.05). Despite issues of reliability for some of the UKB tests, their average loadings on the genomic g factor are substantial, and they have the same average loading of 0.66 on genomic g as the GenLang tests. The residual genetic variances of the 12 cognitive tests after removing the variance explained by genomic g are also significant, ranging from 0.15 for Fluid to 0.93 for Reaction.

On average, genomic g accounted for just under half of the genetic variance for the 12 tests (46.8%). Specific genetic variance, that is, genetic variance not attributed to genomic g, on average, makes up slightly more than half (53.2%) of the genetic variance for the 12 tests. The proportion of variance accounted for by genomic g varies substantially across the 12 tests; nevertheless, the average is comparable for the five tests from GenLang (44.3%), and seven tests from UKB (48.6%).

### SNP Heritabilities of Cognitive Tests are Almost as High Corrected for g as Uncorrected for g

**Fig. 2:**
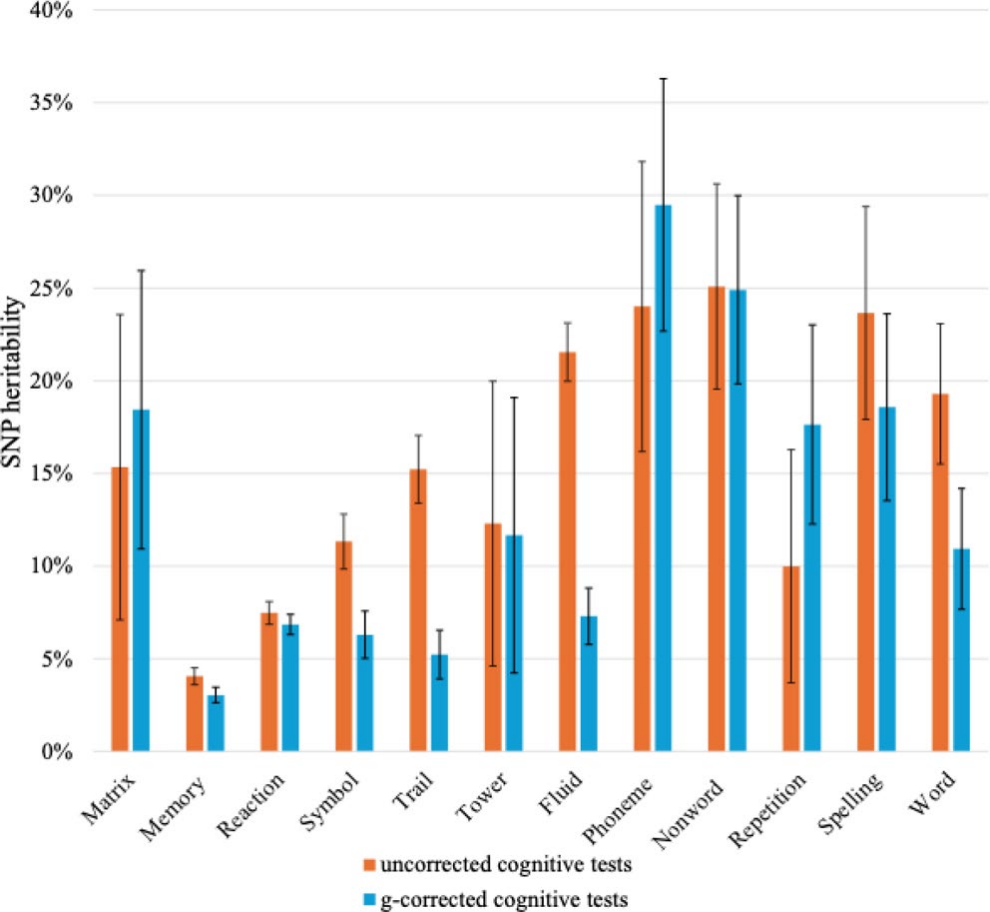
SNP heritabilities for cognitive tests uncorrected for g (orange) and g-corrected (blue) with 95% confidence interval error bars

SNP heritabilities are nearly as high for g-corrected cognitive tests as for the uncorrected cognitive tests (Fig. 2 and Supplementary Table S5): the average SNP heritability is 0.16 (SE = 0.02) for the uncorrected tests and 0.13 (SE = 0.02) for the g-corrected cognitive tests. Comparing SNP heritabilities for the cognitive tests uncorrected and corrected for g reveal some interesting differences across tests. For example, the SNP heritability of Fluid is 0.22 (SE = 0.01) uncorrected for g and 0.06 (SE = 0.01) corrected for g, suggesting that much of the SNP heritability of Fluid is due to g, which is not surprising because Fluid loads highest on genomic g (Fig. 1). In contrast, high SNP heritabilities are also found for Phoneme (0.24, SE = 0.04) and Nonword (0.25, SE = 0.03), but these SNP heritabilities are not diminished for g-corrected cognitive tests (0.29, SE = 0.03 and 0.25, SE = 0.03, respectively). Three tests show significant decreases in SNP heritability before and after g-correction: Memory, Symbol, Trail, Fluid and Word.

### Genetic Correlations among Cognitive Tests before and after g-Correction

**Fig. 3:**
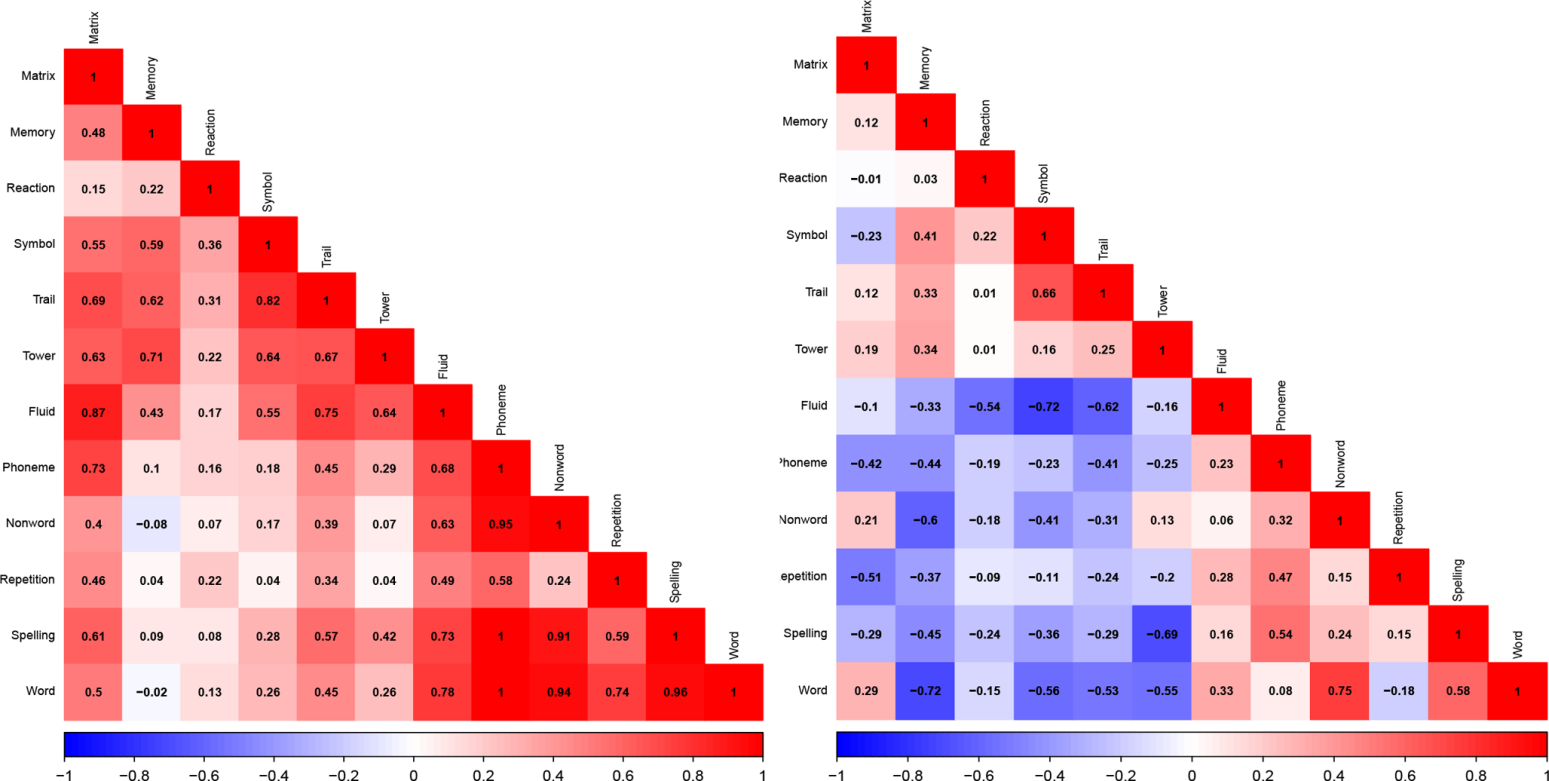
Genomic correlational matrix between cognitive tests before (left) and after (right) g correction

To further investigate the specificity of the 12 tests, we used LDSC in Genomic SEM to estimate the genetic correlations among the 12 tests residualized and un-residualized for genomic g (Fig. 3 and Supplementary Tables S3 and S4).

The un-residualized test scores are positively correlated genetically with each other (mean genetic correlation = 0.45; mean SE = 0.09). On average, the highest correlations are found for tests correlated with Fluid (mean = 0.61), Phoneme (0.57), Matrix (0.55), and Trail (0.55); the lowest correlations are found for tests correlated with Reaction (0.19), Memory (0.29), and Repetition (0.34). The seven tests from UKB are genetically correlated highly positively with one another (mean = 0.59), as are the five tests from GenLang (mean = 0.84), but as expected, the average genetic correlation between tests from the largely nonverbal UKB tests and the verbal GenLang tests is more modest (mean = 0.32).

After residualizing the 12 tests for the genetic effects of g, the positive manifold disappears. The average correlation among the g-corrected cognitive tests is -0.07 (mean SE = 0.09); the average decrease in genetic correlation from before to after g correction is 0.53. After correcting for genomic g, over half of the genetic correlations change from positive to negative, with some correlations between cognitive tests switching from highly positive to highly negative. For example, the greatest switch from positive to negative genetic correlations is for Fluid and Trail, which switched from 0.75 (SE = 0.04) uncorrected for g to -0.62 (SE = 0.09) after g correction. Fluid and Trail are both UKB tests, but this switch from positive to negative is also observed between tests across the two batteries. For example, the genetic correlation between Matrix (UKB) and Repetition (GenLang) changes from 0.46 (SE = 0.20) to -0.51 (SE: 0.15), and from 0.42 to -0.69 for Tower (UKB) and Spelling (GenLang).

**Fig. 4:**
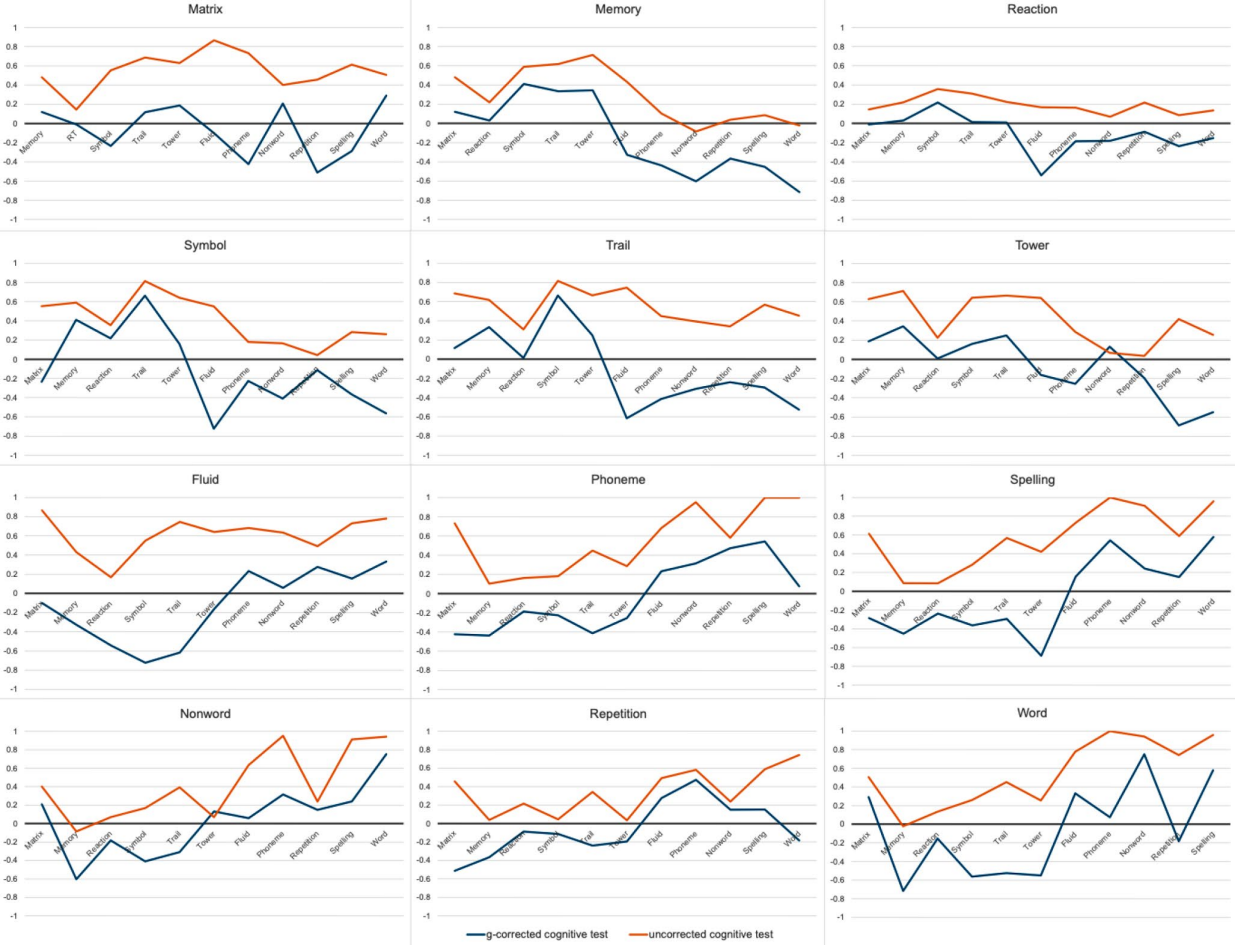
Genetic correlations for each cognitive test with the other 11 cognitive tests uncorrected (orange) and corrected (blue) for genomic g

Although the correlations among the g-corrected cognitive tests are lower than those between the g-corrected tests, the extent to which the correlations decrease is not uniform. Figure 4 highlights the differences in profiles of genetic correlations before and after residualizing g by showing the genetic correlations between each of the 12 tests with the other 11 tests, comparing correlations uncorrected for genomic g (orange line) and corrected for genomic g (blue line). The figure shows the general decrease in genetic correlation after g-correction, as the blue lines are consistently lower than the orange lines. However, the differences in genetic correlations before and after correction across each test are not uniform; the blue and orange lines are not parallel. For instance, Trail shows a relatively small difference (0.16) with Symbol before g-correction (0.82, SE = 0.06) and after g-correction (0.66, SE = 0.09). Trail and Fluid yield the greatest difference (-1.37), going from highly positive (0.75) to highly negative (-0.62).

Importantly, these differences are not merely a function of the extent to which the tests load on genomic g. For instance, Matrix and Trail load 0.84 on genomic g, but their genetic correlations with Word are substantially different before and after correcting for genomic g. The change in correlation between Matrix and Word before and after residualizing g is modest, whereas the change in correlation between Trail and Word is substantial.

We tested the effect of g-loadings more systematically by correlating, for each of the 66 pairs of 12 tests, their difference in their loadings on genomic g (Fig. 1) with their difference in genetic correlations before and after residualizing g (Fig. 3). The resulting correlation was − 0.03, which supports our conclusion that the profile of differential correlations is not due to differential loadings of the tests on genomic g.

### Genetic Correlations with External Traits

To further examine changes in the genetic landscape of cognitive tests after g-correction, we used LDSC to generate correlations for each test before and after correcting for g versus 64 ‘external’ traits for which GWA summary statistics were available. These traits span seven domains: behavior, height and weight, cognition, mental health, personality, physical health, and SES. The genetic correlations are listed in Supplementary Table S27 and shown in Supplementary Figures S10–S28.

Similar to the pattern observed in Fig. 4 among cognitive tests before and after g-correction, genetic correlations with external traits show a similar pattern of attenuation after g-correction, although the overall correlations are much lower in magnitude than the genetic correlations among the cognitive traits. As shown in the genetic correlation matrices before and after g-correction in Fig. 4, the results provide evidence for both genomic g and genomic specificity. Genetic correlations between tests and external traits decline when the tests were corrected for g, indicating a contribution of genomic g. For example, ADHD yields significant negative correlations with most tests, but after g-correction, the genetic correlations are near zero, which suggests that genetic correlations between ADHD and cognitive traits are largely due to genomic g. Specificity is indicated by non-uniform changes in genetic correlations before and after g correction, suggesting a different genetic landscape for g-corrected cognitive tests across external traits. For example, the genetic correlation between ADHD and Trail, a test of executive function, switches from negative (-0.21) to positive (+ 0.20) after g-correction. Another example is that the genetic correlation between the Memory test and educational attainment shifts from positive (0.10) to negative (-0.23) after g-correction. A more detailed summary of these results can be found in Supplementary Note p. 12.

## Discussion

Much of our understanding of the genetics of diverse tests of cognitive ability reflects general cognitive ability (g) rather than the tests themselves. Our results indicate that the genetic landscape of cognitive tests is transformed after controlling for genomic g.

### Cognitive Tests Independent of g (g-Corrected Cognitive Tests) are Heritable

If genetic influences on cognitive tests were entirely due to g, there would be little value in trying to investigate the genetics of g-corrected cognitive tests. For this reason, it is important to note that the heritability of cognitive tests does not merely reflect g. Despite removing almost half of the genetic variance due to g from each of the 12 tests, the average SNP heritability only decreased from 16% before correction for g to 13% after correction. The substantial SNP heritability of g-corrected cognitive tests should encourage more large-scale GWA studies focusing on the genetic specificity of specific cognitive abilities controlling for genomic g.

### The Genetic Landscape of Cognitive Tests Transforms after g-Correction

By correcting the 12 cognitive tests for their covariance, the initial positive manifold observed (average genetic correlation = + 0.45) disappeared. This resulted in an average genetic correlation among the g-corrected cognitive tests of -0.07, which was not statistically significant from zero. However, beyond the disappearance of the positive manifold, the genetic landscape of cognitive tests changed dramatically after g-correction.

Uncorrected for g, many peaks of positive genetic correlations emerge -- 29 of the genetic correlations are 0.50 or greater and six are greater than 0.90 -- and the lowest genetic correlations are near zero. Corrected for g, the genetic landscape of cognitive tests is hillier, with peaks of positive genetic correlations between g-corrected cognitive tests as high as 0.66 (between Symbol and Trail) and deep valleys of negative correlations as low as -0.72 (between Fluid and Symbol and between Memory and Word). The large negative correlations suggest that many SNPs associated with these pairs of tests have effects in opposite directions when g is controlled. This finding suggests that GWA analyses of g-corrected cognitive tests could yield very different results than those of cognitive tests uncorrected for g, which muddle SNPs associated in one direction with g and SNPs associated in the opposite direction for the g-corrected cognitive tests.

The variability in correlations across test pairs before and after g-correction highlights the complexity of the changes. On average the genetic correlation among test pairs decreased by about 0.50, however there was substantial heterogeneity. Some test pairs shifted from highly positive to strongly negative, such as Fluid and Trail (from + 0.75 to -0.62), Fluid and Reaction (+ 0.75 to -0.62) and Matrix and Phoneme (+ 0.73 to -0.42). In contrast, other pairs changed very little after g-correction, for instance Symbol and Reaction (+ 0.36 to + 0.22) and Repetition and Phoneme (+ 0.58 to + 0.47).

The observed changes in genetic correlations are not merely a reflection of the extent to which the cognitive tests load onto g. For instance, both Matrix and Trail have a factor loading of 0.84 onto g, yet their correlations with Word differ significantly after g-correction. The correlation between Matrix and Word remains positive but decreases slightly (+ 0.51 to + 0.29), whereas the correlation between Trail and Word shifts from positive to negative (+ 0.45 to -0.53). This demonstrates that the changes in genetic correlations after g-correction are driven by components that go beyond their shared covariance (g) and may reflect unique genetic influences associated with specific cognitive tests.

### Genetic Correlations between Cognitive Tests and External Traits also Change after g-Correction

We explored genetic correlations between specific cognitive tests and 64 external traits spanning seven domains: behavior, height and weight, cognition, mental health, personality, physical health, and socioeconomic status. g influences not only the relationships among cognitive tests but also their associations with external traits. By comparing genetic correlations before and after g-correction, we aimed to uncover novel insights with implications that extend beyond cognition, which includes applications in mental health and socioeconomic research, as well as practical relevance to clinical practice.

Overall, genetic correlations with external traits decreased for g-corrected cognitive tests compared to their uncorrected counterparts. Except for Memory and Reaction, g-corrected cognitive tests showed a higher proportion of non-significant correlations across domains. However, overlapping confidence intervals for traits in the behavior, body mass index (BMI), mental health, personality, and physical health domains suggest that these associations are primarily driven by specific genetic components of the cognitive tests, rather than generalist genetic influences captured by g.

There were exceptions to this trend. For instance, before g-correction, most cognitive tests showed negative genetic correlations with ADHD, reflecting its association with lower cognitive performance. After g-correction, the strength of these negative correlations significantly decreased for Matrix, Nonword, Phoneme, Spelling, Symbol, Fluid, and Word, which suggests that these associations are largely attributable to generalist genetic influences. Trail and Memory were notable exceptions, with Memory’s genetic correlation with ADHD becoming significantly positive (+ 0.17) and Trail’s correlation shifting from negative to positive after g-correction.

Clinically, these findings are significant. ADHD is commonly associated with lower performance in cognitive assessments, particularly in processing speed and working memory, which are often used in diagnostic evaluations. However, our results indicate that these associations may primarily reflect generalist genetic influences, with unique components of cognitive tests contributing less to the negative correlation and, in some cases, revealing positive associations. These findings highlight how the removal of generalist genetic influences can uncover unexpected relationships and suggest that the role of specific genetic components in ADHD’s association with cognitive performance warrants further investigation.

### Limitations

Our conclusions are limited in four ways, the first of which relates to the creation and modelling of genomic g. In these analyses, we conceptualized g as the common variance among the 12 cognitive test summary statistics, which we then removed to create g-corrected summary statistics for each test. This approach means that genomic g and therefore the 12 g-corrected summary statistics are inherently limited by the cognitive test summary statistics included. The modelling and therefore controlling of g cannot be perfect, which means that some of the residual variance in the g-corrected summary statistics may reflect variance not uniquely attributable to the cognitive test itself, but g variance that the cognitive tests included did not capture.

To mitigate the impact of the imperfect modelling of g, we aimed to create a comprehensive measure of g, and combined summary statistics of both verbal and nonverbal cognitive tests: five verbal cognitive test summary statistics from GenLang (Eising et al. 2022), and seven primarily nonverbal cognitive test summary statistics from UKB (de la Fuente et al. 2021). However, differences in assessment across the two studies could have influenced the results. UKB participants were all assessed using the same tests, whereas GenLang involved a meta-analysis of 22 studies that included a wide range of cognitive tests. In addition, participants in UK Biobank were adults aged 40–75, whereas GenLang participants were aged 5–26 but were mostly in late childhood. These differences in test design and age ranges could have contributed to the observed differences in average genetic correlations. For instance, the average genetic correlation among the seven largely nonverbal UKB tests dropped from 0.59 before g correction to 0.00 after correction, whereas the five verbal GenLang tests decreased from 0.84 to 0.31. These differences likely reflect the greater similarity among the verbal tasks in GenLang compared to the more varied mostly nonverbal tasks in UKB. Additionally, these differences may account for the poor model fit of the common factor model within Genomic SEM.

However, despite these differences, our most striking results of swings from positive genetic correlations for cognitive tests to negative genetic correlations for g-corrected cognitive tests emerged not just between UKB and GenLang but also within each dataset. Furthermore, previous analyses of genetic correlations for IQ across childhood, adulthood and older adulthood have yielded genetic correlations of 0.86 (SE = 0.11) between childhood and adulthood and 0.67 (SE = 0.24) between adulthood and older adulthood (Savage et al. 2018). Such findings support our approach of combining summary statistics obtained from cognitive tests conducted by participants of different ages. Additionally, the summary statistics for genomic g correlated 1 (rG = 1.01; SE = 0.03) with the summary statistics for intelligence (IQ3; Savage et al. 2018), and also correlated highly with other g-related external traits (Supplementary Tables S28 and 29). These results further support the validity of our conceptualization and modelling of g. Nonetheless, what is needed is an even larger UKB-type study of the same individuals tested at the same ages on the same broad battery of tests of cognitive ability, which might be possible, for example, with the 5 million participants targeted for Our Future Health (https://ourfuturehealth.org.uk/).

A second limitation is conceptual: our analyses of g-corrected cognitive tests are intrinsically circular in that they are based on the effect sizes of SNPs from GWA analyses of cognitive tests uncorrected for g. Genomic SEM makes it possible to use these summary SNP statistics to conduct GWA analyses of g-corrected cognitive tests independent of g by statistically correcting for the general effects of g. However, direct GWA analyses of g-corrected cognitive tests seem likely to yield more associations specific to cognitive tests. The heritability of g-corrected cognitive tests should encourage these studies. However, the challenges to conducting large GWA studies of g-corrected cognitive tests seem daunting, especially the requirement of psychometrically strong measures of verbal and nonverbal abilities. It would be ideal to assess g using the same measures, and for this reason, a freely available 15-minute gamified measure of g and verbal and nonverbal abilities with excellent psychometric properties has been developed in the hope that it can be incorporated in new and existing GWA studies (Malanchini et al. 2021).

A third limitation is general to all GWA analyses: missing heritability. Although SNP heritabilities and twin heritabilities are higher for cognitive abilities than for other behavioral domains, SNP heritabilities are less than half the twin heritabilities and variance predicted by polygenic scores is half the SNP heritabilities (Plomin and von Stumm 2018). For now, the most likely strategy for increasing the predictive power of polygenic scores is ever-larger GWA studies with better measures of cognitive abilities and g, but technological advances such as whole-genome sequencing and artificial intelligence offer hope for additional strategies.

Another strategy is to conduct more large-scale multi-ancestry GWA studies that not only increase statistical power, but also allow for genomic discoveries beyond European populations. This leads to the fourth limitation of this study, which is that the GWA summary statistics used are derived exclusively from white participants of European ancestry, which reduces the representativeness of the sample and limits the generalizability of our findings to other populations. This approach was chosen because most GWA studies of cognitive tests rely on participants with European ancestry. To address this limitation, more genetic and genomic studies of under-represented ancestries are needed. Specifically, large-scale, multi-ancestry GWA studies are needed to identify genetic variants shared among different ancestries, as well as those unique to a particular ancestry.

### Implications

The main implication of these findings about the changing genetic landscape of cognitive tests after controlling genomic g is that if research on cognitive tests in education, neuroscience and genetics does not control for g, the same g variance will be investigated repeatedly in the guise of verbal, spatial, memory and other cognitive abilities. Removing the pervasive influence of genomic g is necessary to investigate the genetic specificity of specific cognitive abilities. To foster this new direction for research, the summary statistics from our genome-wide association analyses of 12 g-corrected cognitive tests are freely available on the GWAS Catalogue (https://www.ebi.ac.uk/gwas/) to be used by researchers to create polygenic scores that focus on the specificity of cognitive tests.

## Electronic Supplementary Material

Below is the link to the electronic supplementary material.

Supplementary Material 1

Supplementary Material 2

## Data Availability

No datasets were generated or analysed during the current study.
