# Supplementary material for: The Genetic Specificity of Cognitive Tests After Controlling for General Cognitive Ability
Source: Behav Genet. 2025 Feb 18;55(2):103–13. doi: 10.1007/s10519-025-10213-5 (PMC11882725; doi:10.1007/s10519-025-10213-5)
Supplement: Supplementary file 1 — Supplementary Material 1 [file 10519_2025_10213_MOESM1_ESM.docx]

**Supplementary Notes and Figures**

**The genetic specificity of cognitive tests**

**after controlling for general cognitive ability**

(2024)

Francesca Procopio^1^, Engin Keser^1^, Jacob Knyspel^1^, Margherita Malanchini^1,2^, Kaili Rimfeld^1,3^, Robert Plomin^1^

^1^ Social, Genetic and Developmental Psychiatry Centre, Institute of Psychiatry, Psychology and Neuroscience, King’s College London, London, United Kingdom

^2^ School of Biological and Chemical Sciences, Queen Mary University of London, London, United Kingdom

^3^ Department of Psychology, Royal Holloway, University of London, Egham, United Kingdom

**Contents**

**Background and methods**

**Explanation of genomic and phenotypic data** ……...………………….………..…… 2

UK Biobank and GenLang Consortium

**Data collection procedures** ……………...………………………..…..…….…….….…. 3

UK Biobank and GenLang Consortium

**Sample size and sample size rationale** ……...…………………….….………………. 4

**UKB and GenLang GWA summary statistics** ……………………………...…….…... 5

**Genomic SEM** ……………………………………………………………………....….…... 8

**Effective sample size** …………………………………………………………….…..…… 9

**GWA significant hits and functional mechanisms** ……………..…………….….… 10

**OSF deviations** ………………………………..………………………………….…….... 11

**Results**

**Genetic correlations with external traits** …….………………...………….………… 12

**Figures**

**Supplementary Figure S1 to S8** ………………………………………………………. 14

Manhattan plots for GWA analyses of four cognitive tests corrected and uncorrected for genomic g

**Supplementary Figure S9** ……………………………………………………………… 18

Manhattan plot for GWA analysis of genomic g

**Supplementary Figure S10 to S23** ………………………………………….…..……. 19

Genetic correlations for the cognitive tests before and after g-correction with external traits

**Supplementary Figure S24 to S27** ……………………………………………………. 33

Genetic correlations with external traits for genomic g

**References** …………………………………………………………………………….…..……. 37

**Explanation of genomic and phenotypic data**

In order to create a more representative g, which includes both verbal and nonverbal tests, we used GWA summary statistics from two studies: de la Fuente et al., (2021), which primarily involved nonverbal performance tests, and Eising et al. (2022), which focused on reading and language tests. The first study used phenotypic and genomic data from the UK Biobank (UKB; (Sudlow et al., 2015). The UKB is one of the world’s largest biobanks; from 2006 to 2010, it recruited half a million individuals aged between 40 – 69 years from across the UK. The UKB GWA study used genotypic data from UKB participants of European ancestry who completed at least one of the seven cognitive tests administered by the UKB. The sample included 332,050 unrelated individuals of European descent, aged 40 – 70 in the first wave of assessments and 45 – 75 in the second wave of assessments. The UKB paper detailed the UKB’s genotyping procedures and the univariate GWA analyses conducted for each of the seven cognitive tests: Matrix Pattern Recognition (a measure of nonverbal reasoning, which we refer to as ‘Matrix’), Pairs Matching Test (episodic memory, ‘Memory’), Reaction Time (perceptual motor speed, ‘Reaction’), Symbol Digit Substitution (information processing speed, ‘Symbol’), Trail Making Test-B (executive function, ‘Trail’), Tower Rearranging (executive function, ‘Tower’), and Verbal-Numerical Reasoning (fluid intelligence, ‘Fluid’).

The GenLang consortium meta-analyzed GWA summary statistics for five verbal domains: Word Reading (number of words read aloud correctly, ‘Word’), Nonword Reading (nonwords are phonemes that look like words but have no meaning, ‘Nonword’), Spelling, Phoneme Awareness (‘Phoneme’), and Nonword Repetition (number of nonwords repeated aloud correctly, ‘Repetition’). GenLang analyzed data from 22 independent cohorts, with total sample sizes from 12,000 to 30,000 (aged between 5 to 26 years), for the five domains (Eisling et al., 2022). More details about the cohorts and measures used by each cohort can be found in the paper’s supplementary material and the GenLang Consortium website: https://www.genlang.org/index.html.

**Data collection procedures**

Details of the data collection procedures are available for UKB (Bycroft et al., 2018; Fawns-Ritchie & Deary, 2020) and GenLang (Eising et al., 2022).

UKB collected cognitive data across multiple waves of data collection. At baseline, 2006 – 2010, cognitive data was collected from the majority of participants for three of the cognitive assessments included in this project: Memory (N = 331,679), Reaction (N = 330,024) and Fluid (N = 171,304). The sample size of the fluid is substantially lower than the other two measures as it was only added to the baseline participant assessment part-way through the assessment wave. A subsample of about 20,000 UKB participants underwent repeated testing for these cognitive tests four years after the baseline assessment (Lyall et al., 2016), but de la Fuente only included data from the baseline assessment in the GWA analyses.

Data for the four other UKB cognitive measures were collected at subsequent UKB data collection waves. The sample sizes of the cognitive tests are substantially lower than the cognitive measures collected at baseline: Symbol (N = 87,741), Trail (N = 78,547), Matrix (11,365) and Tower (N = 11,263).

Unlike UKB, the GenLang summary statistics were the result of meta-analyses of GWA analyses of cognitive data from 22 cohorts. GenLang used cognitive measures that differed across the cohorts in language, testing instrument and age of assessment. To reduce the heterogeneity across the cohorts, GenLang employed several exclusion criteria, such as removing cohorts from their meta-analyses if they deviated more than 4 standard deviations from the mean of each specific phenotype. If phenotypic data were available for participants at multiple ages, they chose the age that was closest to the assessment ages of the largest cohorts.

**Sample size**

The number of participants producing GWA summary statistics varies for the different cognitive tests: from 11,263 to 331,679 in UKB and from 13,633 for phoneme awareness to 33,959 for word reading in GenLang.

| **Cognitive test** | **GWA sample size** |
| --- | --- |
| Matrix *UKB* | 11,356 |
| Memory *UKB* | 331,679 |
| Reaction *UKB* | 330,024 |
| Symbol *UKB* | 87,741 |
| Trails *UKB* | 78,547 |
| Tower *UKB* | 11,263 |
| Fluid *UKB* | 171,304 |
| Phoneme *GenLang* | 12,411 |
| Nonword *GenLang* | 16,746 |
| Repetition *GenLang* | 12,828 |
| Spelling *GenLang* | 17,278 |
| Word *GenLang* | 27,180 |

**Sample size rationale**

The UKB study removed participants with non-British ancestry, high SNP missingness, high relatedness, and sex mismatch between self-reported and genetic data. GenLang meta-analyzed GWA summary level data across the 22 cohorts separately for participants of mixed ancestry and participants of European ancestry only. To be consistent with the UKB summary statistics, we used GenLang GWA summary statistics only from meta-analyses with participants of European ancestry.

**UKB and GenLang GWA summary statistics**

As described above, we used GWA summary statistics for seven cognitive tests from the UKB analyses (de la Fuente et al., 2021) and five cognitive tests from GenLang (Eising et al., 2022).

**UKB and GenLang cognitive measures**

Here we describe the twelve cognitive measures from which the UKB and GenLang GWA summary statistics were derived.

UKB (de la Fuente et al., 2021)

1. Matrix pattern recognition (Matrix)

N = 11,356

The matrix pattern recognition test, a test of non-verbal reasoning, is an adaptation of the Raven’s Progressive Matrices test. Participants were shown a matrix design with a missing piece on a computer screen. The pattern has a logical order, and the participant is asked to complete the matrix by selecting one of 6 to 8 puzzle pieces. There were 2 practice matrices and 13 test matrices that ranged in difficulty from easy to difficult. The score was the number of matrices correctly solved in 3 minutes.

1. Memory pairs-matching test (Memory)

N = 331,679

Visual declarative memory was assessed during the baseline assessment using a task that involved matching pairs of symbols. In this self-administered task, the participants were shown 12 cards with pairs of matching symbols for 5 seconds. Each of the 12 cards consisted of 6 matching pairs of symbols. After 5 seconds, one of the paired symbols was hidden, and the participants had to select from memory the location of the corresponding symbols. The task did not have a time limit, and the score was the number of correct items.

1. Reaction time (Reaction)

N = 330,024

Reaction time, which assesses processing speed, was one of the three cognitive tests administered during two assessment visits; scores from the first assessment with the larger sample size were used in the GWA analysis. In each trial, participants were shown 2 cards. If the cards were identical, the participants pressed a button as quickly as possible; if the cards had different symbols, they were to do nothing. After 5 practice trials, there were 7 test trials in which 4 trials showed cards with matching symbols. The score is the mean time in milliseconds to press the button for the 4 test trials. The final score was multiplied by minus 1, so that a higher score indicated better performance.

1. Symbol digit substitution test (Symbol)

N = 87,741

Participants were shown a key, which graphically illustrated pairs of shapes and numbers. Below the key was a row of shapes, and the participants were asked to use the key to enter the corresponding number for each shape. The score was the number of correct matches made in one minute.

1. Trail making test-B (Trail)

N = 78,547

Another perceptual speed test, a self-administered computerized version of the Halstead-Reitan Trail Making test, was administered during the neuroimaging visit and the web-based cognitive assessment. Participants were shown the numbers 1-13, and the letters A-L arranged randomly on a computer screen. The participants were instructed to switch between touching numbers and letters in ascending order (1-A-2-B-3-C etc.) as quickly as possible. The score was the time it took in seconds to successfully complete the two sections of the test. The scores were multiplied by minus 1 so that higher scores indicated better performance.

1. Tower rearranging (Tower)

N = 11,263

Another nonverbal reasoning test is the Tower rearranging test, self-administered during the neuroimaging visit. The participants were shown a display (Display A) with three pegs and three differently coloured hoops on the three pegs. A second display (Display B) was shown below, which also had three pegs, but had the three coloured hoops arranged differently. The participant’s task was to calculate the number of moves it would take to change Display A to match Display B. The score was the number of correctly completed trials in 3 minutes.

1. Verbal-Numerical reasoning test (Fluid)

N = 171,304

This test is a numerical reasoning test administered in a 13-item multiple-choice paper-and-pencil form in a first visit and administered again at a separate visit in a web-based version which included an extra item. Scores from the first assessment were used in the GWA analysis. The score was the number of items answered correctly in 2 minutes.

GenLang (Eising et al., 2022)

1. Phoneme awareness (Phoneme)

Meta-analysis total sample: 12 cohorts (N = 13,633)

Meta-analysis European ancestry only: 11 cohorts (N = 12,411)

The summary statistics for phoneme awareness were derived from a meta-analysis of GWA studies of measures which assessed the number of words correctly altered in phenome deletion/elision tasks, as well as spoonerism tasks, in which the participants were asked to swap the first sounds of words.

1. Nonword reading (Nonword)

Meta-analysis total sample: 13 cohorts (N = 17,984)

Meta-analysis European ancestry only: 12 cohorts (N = 16,746)

The summary statistics were derived from a meta-analysis of GWA studies of measures of nonword reading. In the assessments, nonwords had to be read aloud correctly from a list. Some tests were timed; other had no time limit.

1. Nonword repetition accuracy (Repetition; the number of words or phonemes presented orally and repeated correctly)

Meta-analysis total sample: 10 cohorts (N = 14,046)

Meta-analysis European ancestry only: 10 cohorts (N = 12,828)

The summary statistics for nonword repetition accuracy were derived from a meta-analysis of GWA studies of measures which assessed the number of words or phonemes participants correctly repeated after they were presented orally.

1. Spelling words (Spelling)

Meta-analysis total sample: 15 cohorts (N = 18,515)

Meta-analysis European ancestry only: 14 cohorts (N = 17,278)

The summary statistics were derived from a meta-analysis of GWA studies of measures of spelling words. In the tests, the participants were assessed by the number of words correctly spelt orally or in writing after being dictated.

1. Word reading (Word)

Meta-analysis total sample: 19 cohorts (N = 33,959)

Meta-analysis European ancestry only: 18 cohorts (N = 27,180)

The summary statistics were derived from a meta-analysis of GWA studies of measures of word reading. Word reading was assessed as the number of correct words read aloud from a list. Some tests were timed and others had no time limit.

**Genomic SEM: Creating g-corrected cognitive test GWA summary statistics**

To create summary statistics for the 12 cognitive tests residualized by their genomic covariance, genomic g, we used Genomic Structural Equation Modelling (SEM) in R studio (Grotzinger et al., 2019). Genomic SEM combines SEM with factor analysis to explore the genetic relationship between complex traits. It involves two stages. First, a genetic covariance matrix (S), along with its corresponding sampling matrix (V), is estimated. Then, in the second stage, the SEM of choice is fit to the genetic covariance matrix, and the parameters and their standard errors (SE) are estimated.

In order to estimate the matrices, we first used the “munge” function within Genomic SEM on each of the 12 cognitive test summary statistics to convert them into the required format. We limited the summary statistics to SNPs on the HapMap3 reference panel and filtered out SNPs with a minor allele frequency (MAF) < 0.01 and an imputation score (INFO) < 0.9. We used the munged summary statistics to conduct multivariate LD-Score regression (LDSC) on Genomic SEM to obtain the S and V matrices (Bulik-Sullivan et al., 2015). The diagonal of the S matrix consists of the heritabilities (h^2^) of the measured cognitive tests, and the off-diagonals consist of the coheritabilities of the cognitive tests (i.e., the phenotypically standardized genetic correlations). The V matrix contains the squared SEs on the diagonal, and the sampling covariances on the off-diagonal. To run multivariate LDSC, we used the “ldsc” function in Genomic SEM and used European LD scores and weights from the 1,000 Genomes Phase 3 project.

We then fit a ‘user specified’ model on the S and V matrices to create g-corrected cognitive test GWA summary statistics. This model (Figure 1) extracted a single common factor, genomic g, from the 12 GWA summary statistics, while simultaneously residualizing the effect of genomic g from each cognitive test. We ran the model 12 times, correcting each of the cognitive test summary statistics for genomic g individually.

**Effective sample size**

We used the method proposed by Mallard et al. (2022) to calculate the effective sample size of the genomic g and 12 g-corrected cognitive test GWA summary statistics. The estimate is based on the variance of the minor allele frequency and standard error of the effect sizes from the GWA summary statistics. The effective sample sizes are quite similar to the actual sample sizes.

| **g-corrected cognitive test** | **Effective sample size** |
| --- | --- |
| Matrix *UKB* | 11,394 |
| Memory *UKB* | 327,988 |
| Reaction *UKB* | 323,398 |
| Symbol *UKB* | 95,540 |
| Trails *UKB* | 84,937 |
| Tower *UKB* | 11,481 |
| Fluid *UKB* | 73,434 |
| Phoneme *GenLang* | 12,736 |
| Nonword *GenLang* | 17,531 |
| Repetition *GenLang* | 12,963 |
| Spelling *GenLang* | 17,496 |
| Word *GenLang* | 27,467 |

**GWA significant hits and functional mechanisms**

We used the web-based platform Functional Mapping and Annotation of Genome-Wide Association Studies (FUMA) to further explore the g-corrected cognitive test GWA summary statistics (Watanabe et al., 2017).

The SNP2GENE function was used to identify and define GWA-independent significant SNPs, lead SNPs and genomic risk loci (genomic loci). To identify GWA-independent SNPs, we set a p value threshold of ≤ 5 × 10−8 and a LD r^2^ threshold of <0.6 and used the 1000 Genomes Project Phase 3 derived from European populations as our LD reference panel. The lead SNPs were defined as GWA-independent significant SNPs that were independent of each other at an r^2^ threshold of 0.1. Genomic loci were identified by merging LD blocks of the GWA independent SNPs if they were within 250 kilobases (kb) of each other.

Of the 12 g-corrected cognitive test GWA summary statistics we investigated, FUMA identified GWA-independent significant SNPs only for g-corrected Memory, Reaction, Symbol and Fluid ability. The other eight g-corrected cognitive test GWA summary statistics yielded no significant SNPs, and therefore no results. Supplementary Tables S7 to S26 detail the FUMA analyses for the four g-corrected cognitive test GWA summary statistics with significant findings. We also provide Manhattan Plots of both g-corrected and uncorrected Memory, Reaction, Symbol and Fluid ability as comparisons, as well as the Manhattan plot for genomic g (Supplementary Figures S1 to S9).

Supplementary Tables S7 to S10 list the GWA-independent significant SNPs and corresponding lead SNPs for the four g-corrected cognitive tests. Supplementary Tables S11 to S14 provide further information on the GWA-independent significant SNPs using the GWAS catalogue. Supplementary Tables S15 to S18 provide details of the genomic ‘risk’ loci defined by the independent lead SNPs for each of the four g-corrected cognitive tests.

Instead, Supplementary Tables S19 to S26 present the results of gene-set and gene-property analyses conducted using Multi-marker Analysis of GenoMic Annotation (MAGMA) (v1.08) within FUMA. MAGMA mapped the SNPs to within 35kb upstream and 10kb downstream of 17,274 protein-coding genes. Gene-set analysis (Supplementary Tables S15 to S18) was performed using 10,894 gene sets to identify biological pathways and processes associated with the g-corrected cognitive test GWA summary statistics, and gene-property analysis was performed on 53 specific tissue types to explore potential tissue-specific effects of the SNPs.

**Deviations from the preregistration**

This study includes all the genomic downstream analyses originally preregistered on the Open Science Framework ([https://osf.io/9qwbn/](https://eur03.safelinks.protection.outlook.com/?url=https%3A%2F%2Fosf.io%2F9qwbn%2F&data=05%7C02%7Cfrancesca.procopio%40kcl.ac.uk%7Cf6bdd8415558432f8cef08dcbdb14428%7C8370cf1416f34c16b83c724071654356%7C0%7C0%7C638593816751044593%7CUnknown%7CTWFpbGZsb3d8eyJWIjoiMC4wLjAwMDAiLCJQIjoiV2luMzIiLCJBTiI6Ik1haWwiLCJXVCI6Mn0%3D%7C0%7C%7C%7C&sdata=7HSTxKZJ1GsvVFzE%2FFYbSAs57YqCEr2WjKXkbIHJLlc%3D&reserved=0)). However, we made a modification to the preregistered analyses on the construction of the GWA summary statistics of genomic g and g-corrected cognitive tests. Instead of creating GWA summary statistics of g-corrected cognitive tests through two separate Genomic SEM models (common factor model and then GWAS-by-subtraction) as initially planned, we opted to construct the g-corrected GWA summary statistics using a single model within Genomic SEM. In addition, we did not conduct polygenic score (genome-wide polygenic score, GPS) analyses. We are planning a subsequent project in which we will conduct these analyses.

Finally, upon peer review, we updated the terminology used to address the cognitive phenotypes. We previously referred to the cognitive tests as ‘specific cognitive abilities’ (SCAs), and used the acronym SCA.g to refer to g-corrected specific cognitive abilities.

**Genetic correlations with external traits**

To further examine changes in the genetic landscape of the g-corrected cognitive test GWA summary statistics, we used LDSC to generate genetic correlations for each of the uncorrected and g-corrected cognitive tests versus 64 ‘external’ traits for which GWA summary statistics were available. These traits span seven domains: behavior, height and weight, cognition, mental health, personality, physical health, and socio-economic status (SES). The genetic correlations between the uncorrected and g-corrected cognitive tests with external traits are listed in Supplementary Table S27 and shown in Supplementary Figures S10 – S28.

In the text, we provide an overview of these results, and suggest that the genetic correlations with external traits show a similar pattern of results shown in Figure 4 among the cognitive tests before and after g-correction, although the genetic correlations with external traits are much lower in magnitude than those with the target SCA traits. As in Figure 4, the results of the genetic correlations of the uncorrected and g-corrected cognitive tests with external trats provide evidence for both genomic g and genomic specificity. Here we provide more details about these results.

Across the seven domains, we find a general attenuation in genetic correlations with external traits for the g-corrected cognitive tests compared to the corresponding uncorrected cognitive tests. With the exception of Memory and Reaction, the g-corrected cognitive test GWA summary statistics show a higher proportion of nonsignificant genetic correlations with external traits compared to the uncorrected cognitive tests. Additionally, apart from correlations with traits from the Cognition and SES domain, most 95% confidence intervals for corresponding uncorrected and g-corrected cognitive test GWA summary statistics overlap. Therefore, on the whole, genetic correlations between cognitive tests and traits related to Behavior, BMI-related traits, Mental Health, Personality and Physical Health are largely associated with the specific genetic components of the examined cognitive tests, rather than the generalist component, as captured by genomic g.

However, there are exceptions to this trend. Genetic correlations between ADHD and g-corrected and uncorrected Matrix, Memory, Nonword, Phoneme, Spelling, Symbol, Trail, Fluid, and Word differ significantly. With the exception for Memory, all uncorrected cognitive tests had significant negative correlations with ADHD (Demontis et al., 2019). After g-correction, the genetic correlations for Matrix, Nonword, Phoneme, Spelling, Symbol, Fluid, and Word decrease and are often not significantly different from zero. This suggests that the genetic association between these cognitive tests and ADHD are largely due to genomic g rather than unique genetic effects of the cognitive tests. Trail and Memory deviate from this pattern. The genetic correlation between uncorrected Memory and ADHD is not significant, whereas the g-corrected correlation is significant (+0.17). For Trail, the genetic correlation with ADHD switches from negative to positive after g-correction. This trend also appears with non-p ADHD (Keser et al., 2024); uncorrected Trail correlates negatively, and g-corrected Trails correlates positively. This suggests the relationship between ADHD and SCA is associated with g rather than p, the general factor of psychopathology.

Unlike the correlations with external traits in other domains, most changes in genetic correlations before and after g-correction are significant for traits from the cognitive and SES domains. Given the generalist nature of cognitive traits and their moderate to high genetic correlation with genomic g, it was expected that most correlations would significantly reduce in magnitude, and many would become non-significant. However, we find exceptions to this trend. For instance, the genetic correlations between Memory and Childhood IQ (Benyamin et al., 2014), EA4 (Okbay et al., 2022), and self-reported educational attainment (Davies et al., 2016) shift from positive to negative after g-correction and increased in magnitude.

Finally, all genetic correlations between the cognitive tests and Household income significantly change after g-correction (Hill et al., 2016). Initially, all uncorrected cognitive tests positively correlate with Household income. After g-correction, most correlations attenuate, with some changing direction (Memory, Trail, and Tower). Overall, this indicates that the genomic association with Household income is primarily due to genomic g rather than the unique genetic components of the cognitive tests.


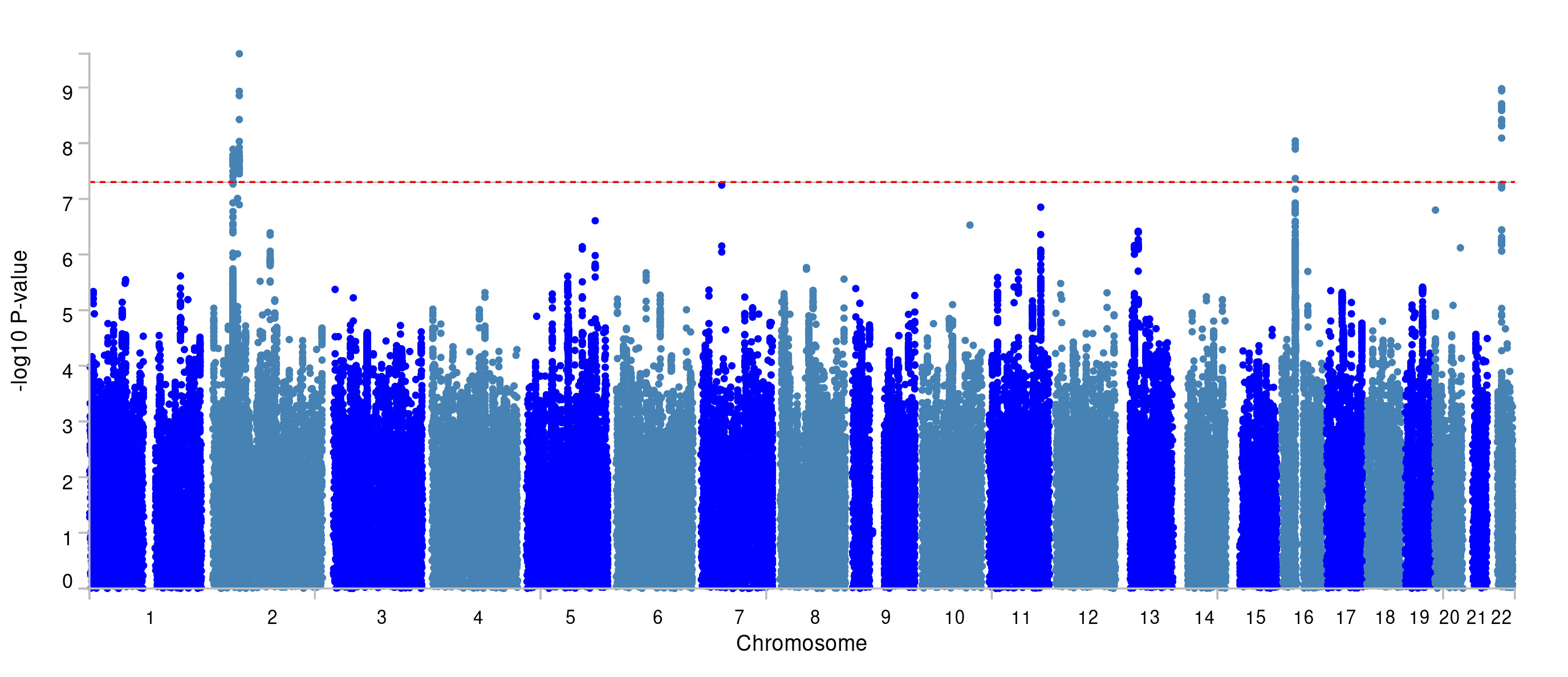
**Supplementary Figure 1:** Manhattan plot of GWA analysis of Memory corrected for genomic g


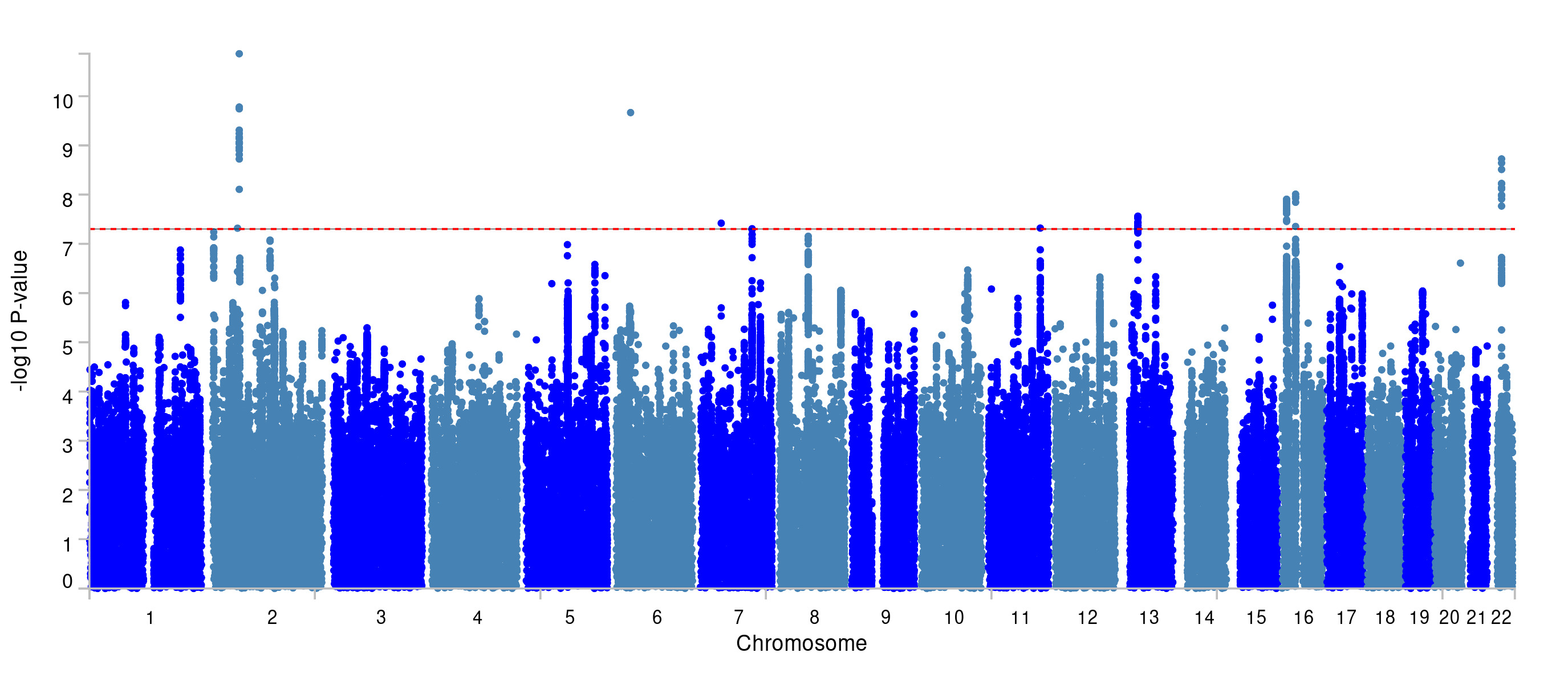
**Supplementary Figure 2:** Manhattan plot of GWA analysis of uncorrected Memory

**Supplementary Figure 3:** Manhattan plot of GWA analysis of Reaction corrected for genomic g


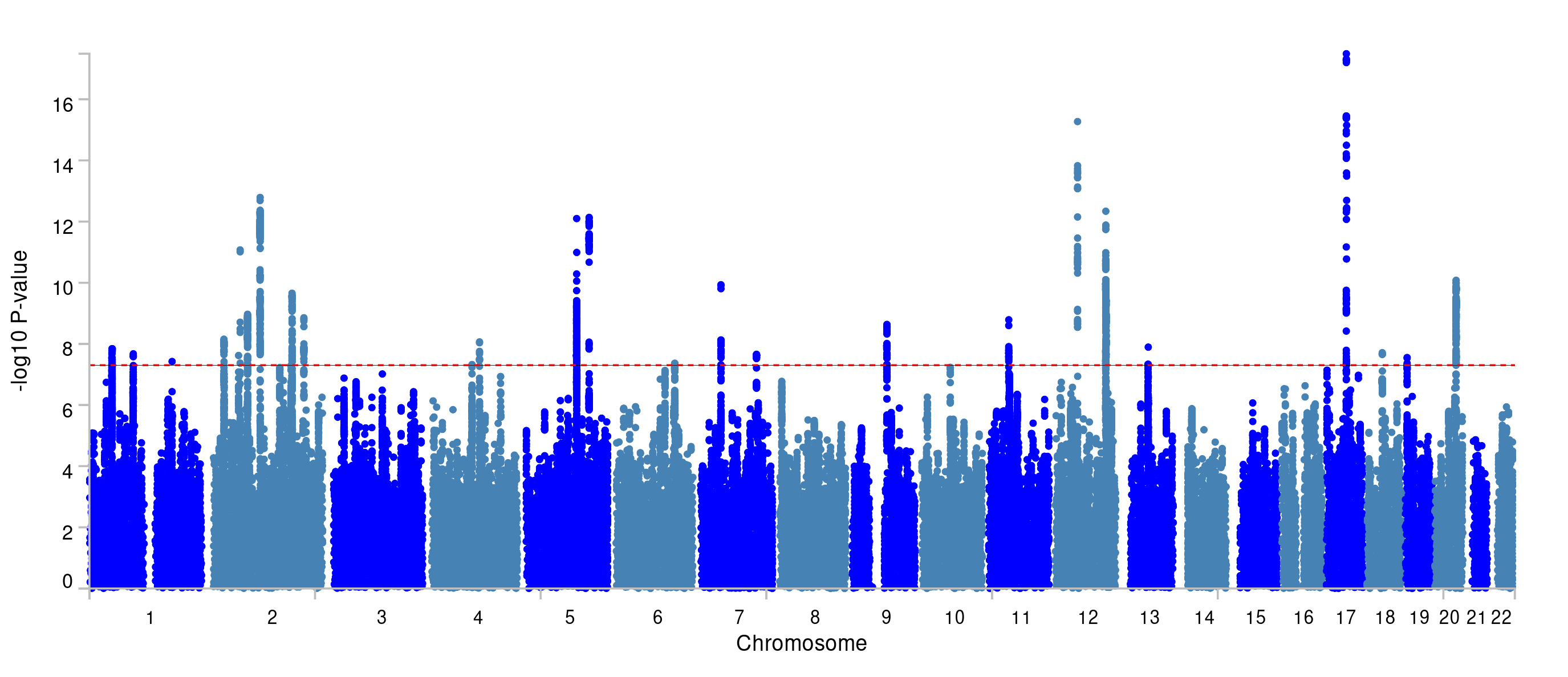


**Supplementary Figure 4:** Manhattan plot of GWA analysis of uncorrected Reaction


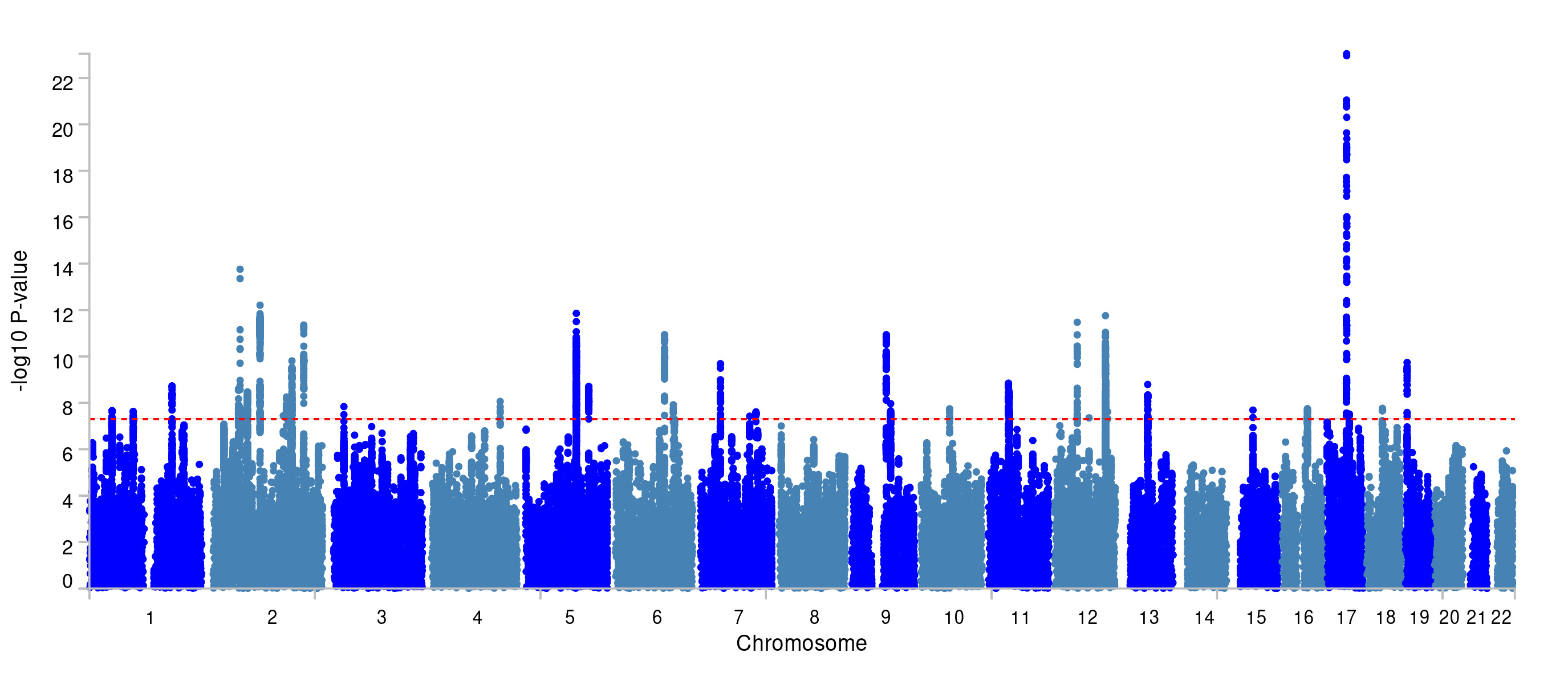


**Supplementary Figure 5:** Manhattan plot of GWA analysis of Symbol corrected for genomic g


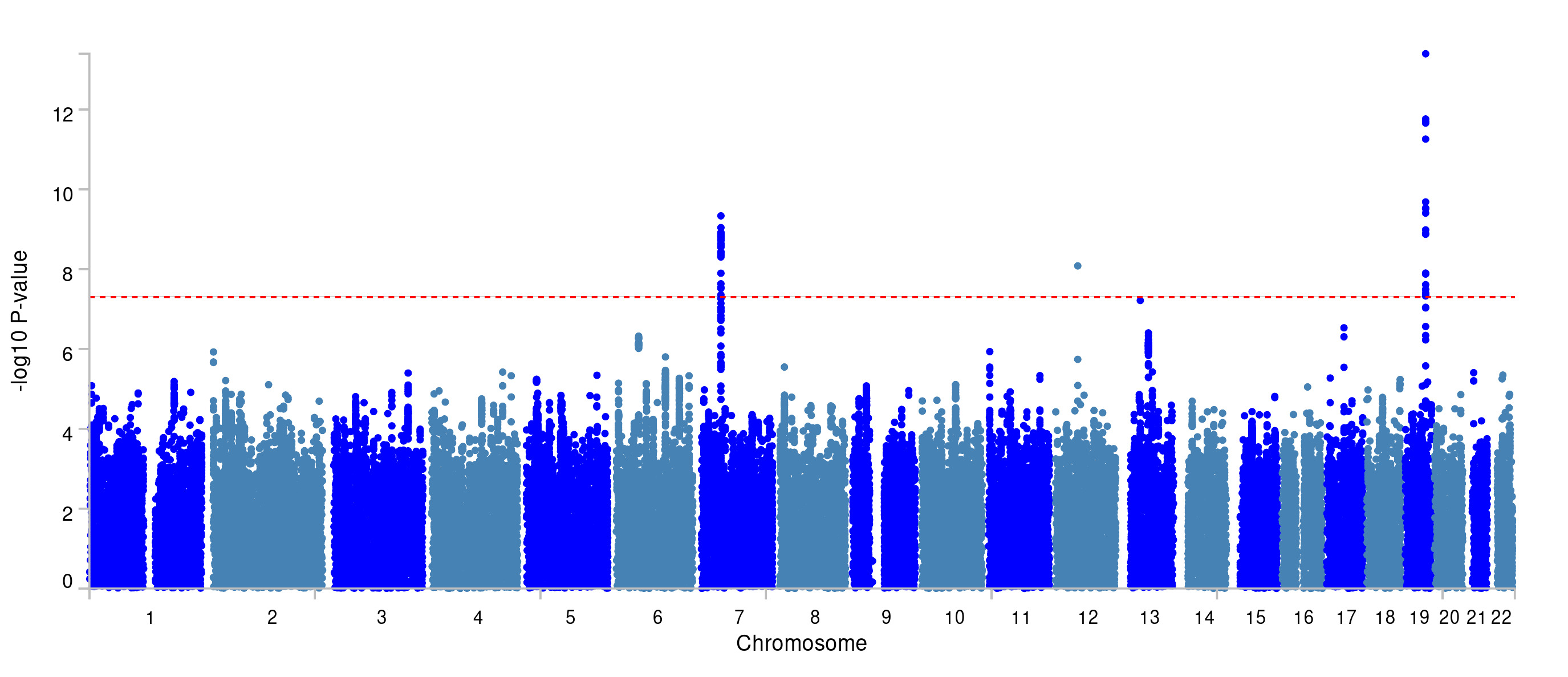


**Supplementary Figure 6:** Manhattan plot of GWA analysis of uncorrected Symbol


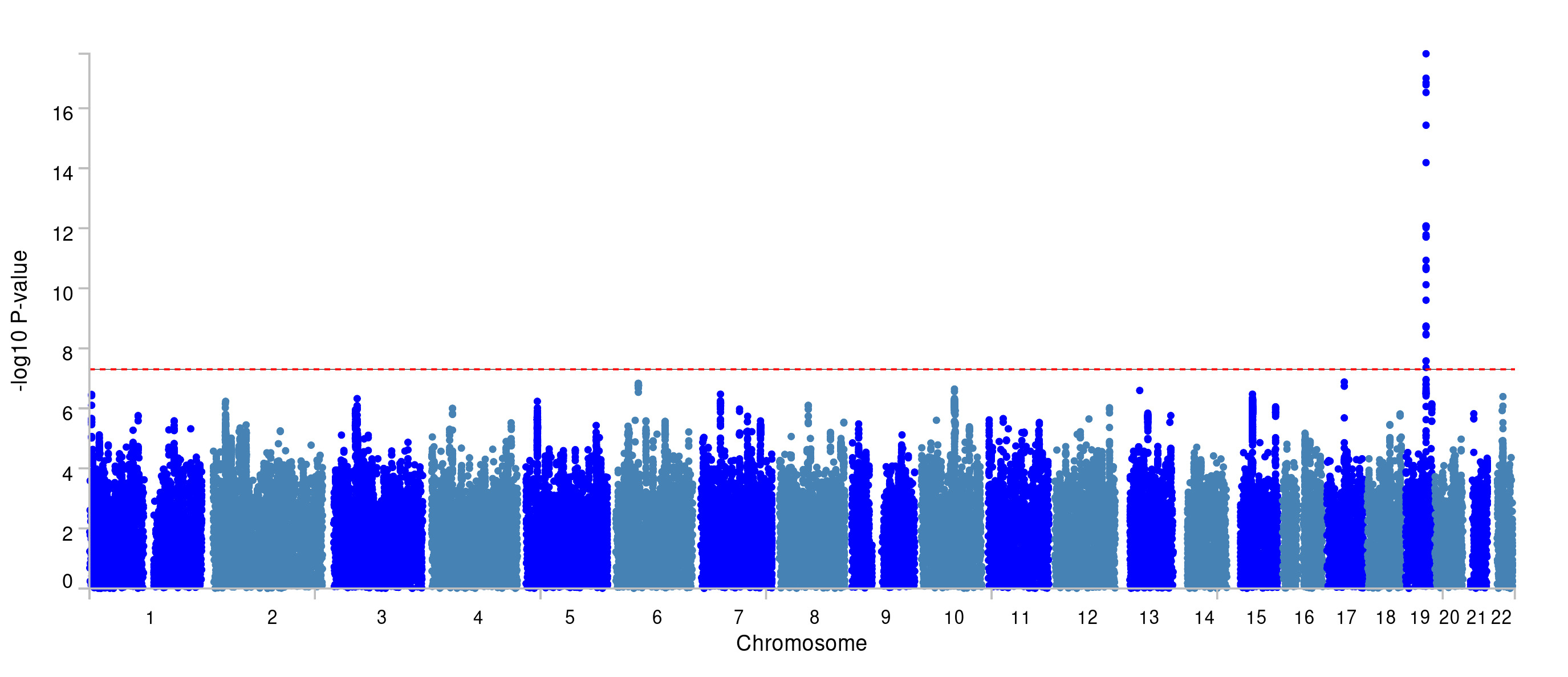


**Supplementary Figure 7:** Manhattan plot of GWA analysis of Fluid corrected for genomic g


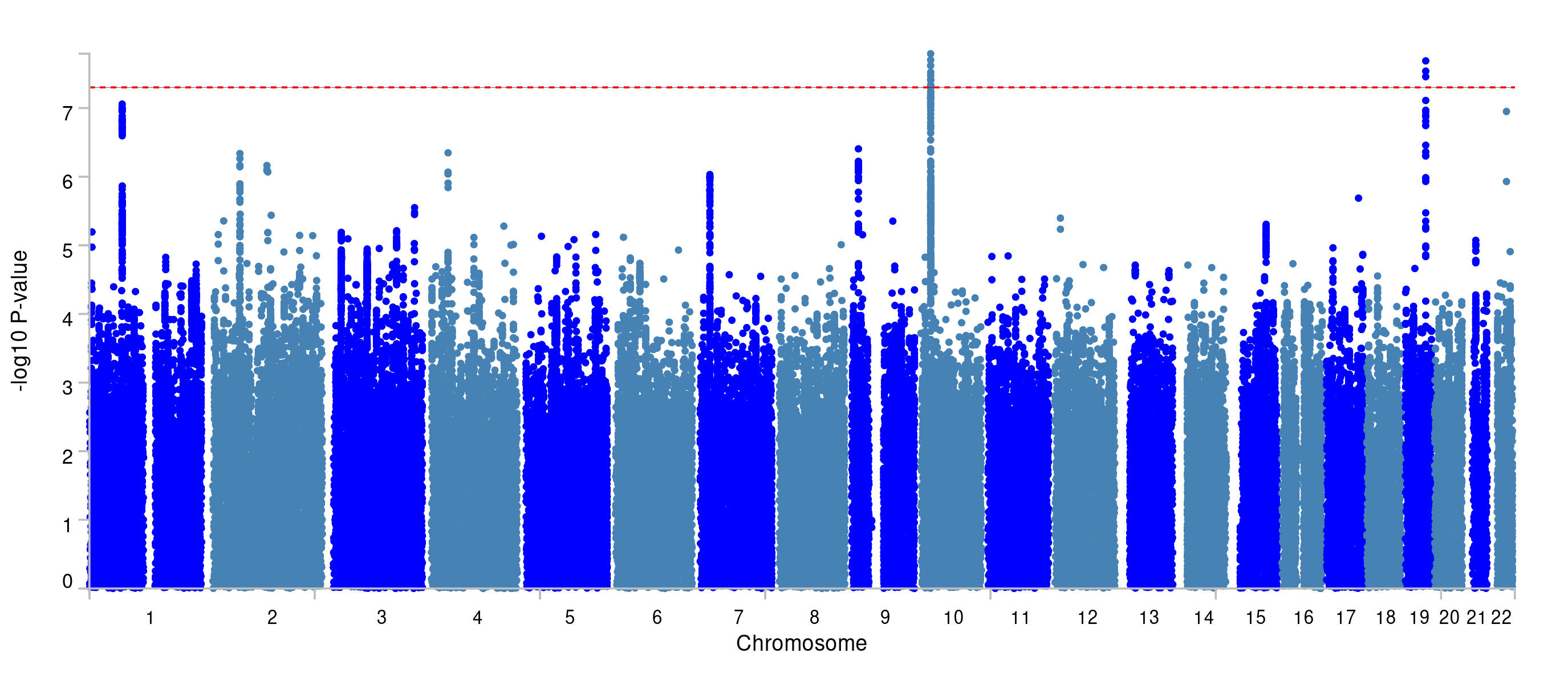


**Supplementary Figure 8:** Manhattan plot of GWA analysis of uncorrected Fluid


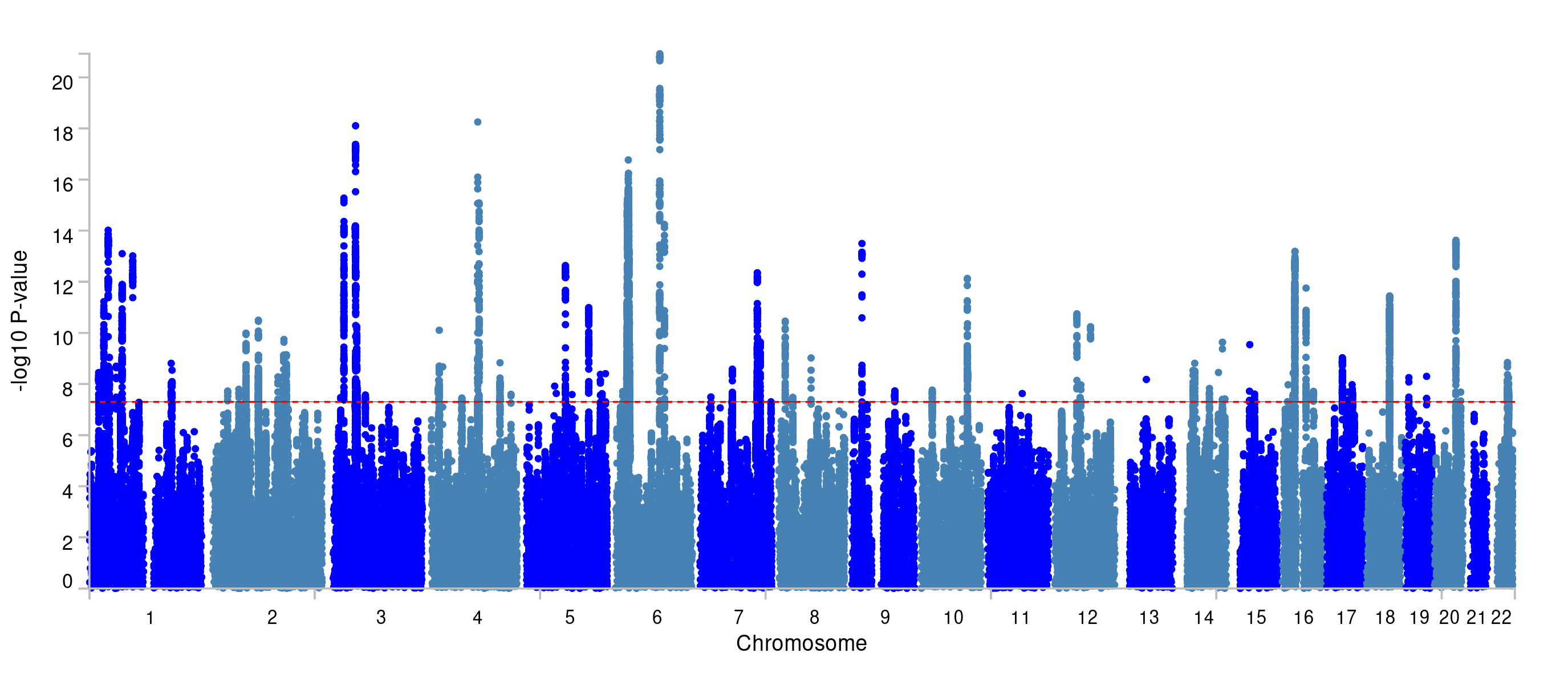


**Supplementary Figure 9:** Manhattan plot of GWA analysis of genomic g


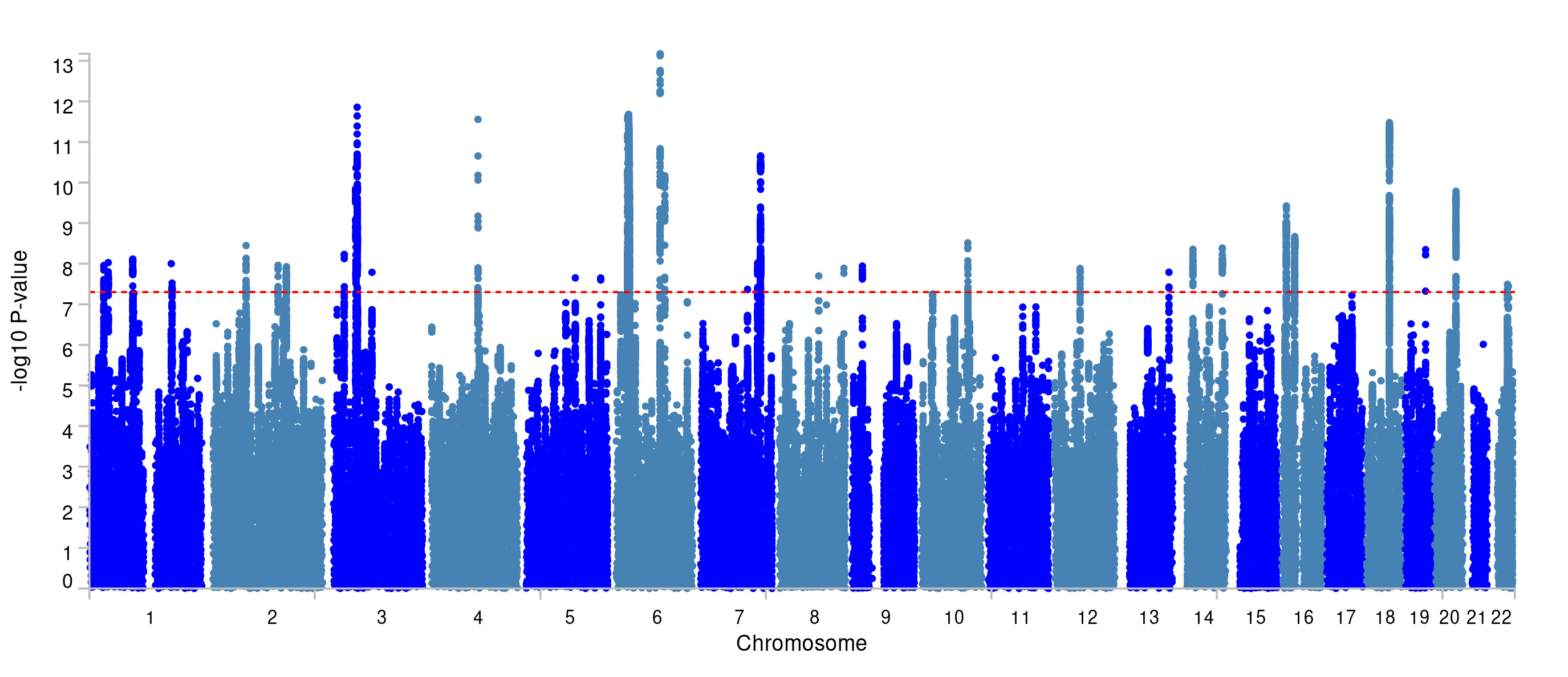

**Supplementary Figure S10:** Genetic correlations between uncorrected (blue) and g corrected (red) Matrix, Memory, Nonword, Repetition, Phoneme and Reaction with external traits in the “Behavior category” with SE error bars

**Supplementary Figure S11:** Genetic correlations between uncorrected (blue) and g-corrected (red) Spelling, Symbol, Trails, Tower, Fluid and Word with external traits in the “Behavior category” with SE error bars

**Supplementary Figure S12:** Genetic correlations between uncorrected (blue) and g-corrected (red) Matrix, Memory, Nonword, Repetition, Phoneme and Reaction with external traits in the “BMI related traits category” with SE error bars

**Supplementary Figure S13:** Genetic correlations between uncorrected (blue) and g-corrected (red) Spelling, Symbol, Trails, Tower, Fluid and Word with external traits in the “BMI related traits category” with SE error bars

**Supplementary Figure S14:** Genetic correlations between uncorrected (blue) and g-corrected (red) Matrix, Memory, Nonword, Repetition, Phoneme and Reaction with external traits in the “Cognitive related traits category” with SE error bars

**Supplementary Figure S15:** Genetic correlations between uncorrected (blue) and g-corrected (red) Spelling, Symbol, Trails, Tower, Fluid and Word with external traits in the “Cognitive related traits category” with SE error bars

**Supplementary Figure S16:** Genetic correlations between uncorrected (blue) and g-corrected (red) Matrix, Memory, Nonword, Repetition, Phoneme and Reaction with external traits in the “Mental Health category” with SE error bars

**Supplementary Figure S17:** Genetic correlations between uncorrected (blue) and g-corrected (red) Spelling, Symbol, Trails, Tower, Fluid and Word with external traits in the “Mental Health category” with SE error bars

**Supplementary Figure S18:** Genetic correlations between uncorrected (blue) and g-corrected (red) Matrix, Memory, Nonword, Repetition, Phoneme and Reaction with external traits in the “Personality category” with SE bars

**Supplementary Figure S19:** Genetic correlations between uncorrected (blue) and g-corrected (red) Spelling, Symbol, Trails, Tower, Fluid and Word with external traits in the “Personality category” with SE bars

**Supplementary Figure S20:** Genetic correlations between uncorrected (blue) and g-corrected (red) Matrix, Memory, Nonword, Repetition, Phoneme and Reaction with external traits in the “Physical Health category” with SE bars

**Supplementary Figure S21:** Genetic correlations between uncorrected (blue) and g-corrected (red) Spelling, Symbol, Trails, Tower, Fluid and Word with external traits in the “Physical Health category” with SE bars

**Supplementary Figure S22:** Genetic correlations between uncorrected (blue) and g-corrected (red) Matrix, Memory, Nonword, Repetition, Phoneme and Reaction with external traits in the “SES category” (Household income and Neighborhood deprivation) with SE bars

**Supplementary Figure S23:** Genetic correlations between uncorrected (blue) and g-corrected (red) Spelling, Symbol, Trails, Tower, Fluid and Word with external traits in the “SES category” (Household income and Neighborhood deprivation) with SE bars

**Supplementary Figure S24:** Genetic correlations between genomic g with external traits in the “Behavior category” with SE bars

**Supplementary Figure S25:** Genetic correlations between genomic g with external traits in the “Cognitive category” with SE bars

**Supplementary Figure S26:** Genetic correlations between genomic g with external traits in the “Mental Health category” with SE bars


**Supplementary Figure S27:** Genetic correlations between genomic g with external traits in the “BMI related traits, Personality, Physical Health and SES traits categories” with SE bars

**References**

Bulik-Sullivan, B. K., Loh, P.-R., Finucane, H. K., Ripke, S., Yang, J., Patterson, N., Daly, M. J., Price, A. L., & Neale, B. M. (2015). LD Score regression distinguishes confounding from polygenicity in genome-wide association studies. *Nature Genetics*, *47*(3), Article 3. https://doi.org/10.1038/ng.3211

Bycroft, C., Freeman, C., Petkova, D., Band, G., Elliott, L. T., Sharp, K., Motyer, A., Vukcevic, D., Delaneau, O., O’Connell, J., Cortes, A., Welsh, S., Young, A., Effingham, M., McVean, G., Leslie, S., Allen, N., Donnelly, P., & Marchini, J. (2018). The UK Biobank resource with deep phenotyping and genomic data. *Nature*, *562*(7726), Article 7726. https://doi.org/10.1038/s41586-018-0579-z

*Childhood intelligence is heritable, highly polygenic and associated with FNBP1L - PubMed*. (n.d.). Retrieved 18 June 2024, from https://pubmed.ncbi.nlm.nih.gov/23358156/

Davies, G., Marioni, R. E., Liewald, D. C., Hill, W. D., Hagenaars, S. P., Harris, S. E., Ritchie, S. J., Luciano, M., Fawns-Ritchie, C., Lyall, D., Cullen, B., Cox, S. R., Hayward, C., Porteous, D. J., Evans, J., McIntosh, A. M., Gallacher, J., Craddock, N., Pell, J. P., … Deary, I. J. (2016). Genome-wide association study of cognitive functions and educational attainment in UK Biobank (N=112 151). *Molecular Psychiatry*, *21*(6), Article 6. https://doi.org/10.1038/mp.2016.45

de la Fuente, J., Davies, G., Grotzinger, A. D., Tucker-Drob, E. M., & Deary, I. J. (2021). A general dimension of genetic sharing across diverse cognitive traits inferred from molecular data. *Nature Human Behaviour*, *5*(1), 49–58. https://doi.org/10.1038/s41562-020-00936-2

Demontis, D., Walters, R. K., Martin, J., Mattheisen, M., Als, T. D., Agerbo, E., Baldursson, G., Belliveau, R., Bybjerg-Grauholm, J., Bækvad-Hansen, M., Cerrato, F., Chambert, K., Churchhouse, C., Dumont, A., Eriksson, N., Gandal, M., Goldstein, J. I., Grasby, K. L., Grove, J., … Neale, B. M. (2019). Discovery of the first genome-wide significant risk loci for attention deficit/hyperactivity disorder. *Nature Genetics*, *51*(1), 63–75. https://doi.org/10.1038/s41588-018-0269-7

Eising, E., Mirza-Schreiber, N., de Zeeuw, E. L., Wang, C. A., Truong, D. T., Allegrini, A. G., Shapland, C. Y., Zhu, G., Wigg, K. G., Gerritse, M. L., Molz, B., Alagöz, G., Gialluisi, A., Abbondanza, F., Rimfeld, K., van Donkelaar, M., Liao, Z., Jansen, P. R., Andlauer, T. F. M., … Fisher, S. E. (2022). Genome-wide analyses of individual differences in quantitatively assessed reading- and language-related skills in up to 34,000 people. *Proceedings of the National Academy of Sciences of the United States of America*, *119*(35), e2202764119. https://doi.org/10.1073/pnas.2202764119

Fawns-Ritchie, C., & Deary, I. J. (2020). Reliability and validity of the UK Biobank cognitive tests. *PLoS ONE*, *15*(4), e0231627. https://doi.org/10.1371/journal.pone.0231627

Grotzinger, A. D., Rhemtulla, M., de Vlaming, R., Ritchie, S. J., Mallard, T. T., Hill, W. D., Ip, H. F., Marioni, R. E., McIntosh, A. M., Deary, I. J., Koellinger, P. D., Harden, K. P., Nivard, M. G., & Tucker-Drob, E. M. (2019). Genomic structural equation modelling provides insights into the multivariate genetic architecture of complex traits. *Nature Human Behaviour*, *3*(5), Article 5. https://doi.org/10.1038/s41562-019-0566-x

Hill, W. D., Hagenaars, S. P., Marioni, R. E., Harris, S. E., Liewald, D. C. M., Davies, G., Okbay, A., McIntosh, A. M., Gale, C. R., & Deary, I. J. (2016). Molecular Genetic Contributions to Social Deprivation and Household Income in UK Biobank. *Current Biology*, *26*(22), 3083–3089. https://doi.org/10.1016/j.cub.2016.09.035

Keser, E., Liao, W., Allegrini, A. G., Rimfeld, K., Eley, T. C., Plomin, R., & Malanchini, M. (2024). *Isolating transdiagnostic effects reveals specific genetic profiles in psychiatric disorders.* (p. 2023.12.20.23300292). medRxiv. https://doi.org/10.1101/2023.12.20.23300292

Lyall, D. M., Cullen, B., Allerhand, M., Smith, D. J., Mackay, D., Evans, J., Anderson, J., Fawns-Ritchie, C., McIntosh, A. M., Deary, I. J., & Pell, J. P. (2016). Cognitive Test Scores in UK Biobank: Data Reduction in 480,416 Participants and Longitudinal Stability in 20,346 Participants. *PLOS ONE*, *11*(4), e0154222. https://doi.org/10.1371/journal.pone.0154222

Mallard, T. T., Karlsson Linnér, R., Grotzinger, A. D., Sanchez-Roige, S., Seidlitz, J., Okbay, A., de Vlaming, R., Meddens, S. F. W., Palmer, A. A., Davis, L. K., Tucker-Drob, E. M., Kendler, K. S., Keller, M. C., Koellinger, P. D., & Harden, K. P. (2022). Multivariate GWAS of psychiatric disorders and their cardinal symptoms reveal two dimensions of cross-cutting genetic liabilities. *Cell Genomics*, *2*(6), 100140. https://doi.org/10.1016/j.xgen.2022.100140

Okbay, A., Wu, Y., Wang, N., Jayashankar, H., Bennett, M., Nehzati, S. M., Sidorenko, J., Kweon, H., Goldman, G., Gjorgjieva, T., Jiang, Y., Hicks, B., Tian, C., Hinds, D. A., Ahlskog, R., Magnusson, P. K. E., Oskarsson, S., Hayward, C., Campbell, A., … Young, A. I. (2022). Polygenic prediction of educational attainment within and between families from genome-wide association analyses in 3 million individuals. *Nature Genetics*, *54*(4), Article 4. https://doi.org/10.1038/s41588-022-01016-z

Sudlow, C., Gallacher, J., Allen, N., Beral, V., Burton, P., Danesh, J., Downey, P., Elliott, P., Green, J., Landray, M., Liu, B., Matthews, P., Ong, G., Pell, J., Silman, A., Young, A., Sprosen, T., Peakman, T., & Collins, R. (2015). UK Biobank: An Open Access Resource for Identifying the Causes of a Wide Range of Complex Diseases of Middle and Old Age. *PLOS Medicine*, *12*(3), e1001779. https://doi.org/10.1371/journal.pmed.1001779

Watanabe, K., Taskesen, E., van Bochoven, A., & Posthuma, D. (2017). Functional mapping and annotation of genetic associations with FUMA. *Nature Communications*, *8*(1), Article 1. https://doi.org/10.1038/s41467-017-01261-5
